# Emergent multiscale organisation of neural dynamics fragments in anaesthesia

**DOI:** 10.1162/IMAG.a.1364

**Published:** 2026-09-17

**Authors:** Borjan Milinkovic, Anil K. Seth, Lionel Barnett, Olivia Carter, Thomas Andrillon

**Affiliations:** Melbourne School of Psychological Sciences, University of Melbourne, Melbourne, Australia; INSERM/Institut du Cerveau (ICM), Hôpital de la Pitié-Salpêtrière, Paris, France; Paris-Saclay Institut des Neurosciences, CNRS, Gif-sur-Yvette, Paris, France; Sussex Centre for Consciousness Science, Department of Informatics, University of Sussex, Brighton, United Kingdom; Canadian Institute for Advanced Research, Program on Brain, Mind, and Consciousness, Toronto, Canada; Monash Centre for Consciousness & Contemplative Studies, Monash University, Melbourne, Australia

**Keywords:** emergence, consciousness, anaesthesia, dimensionality reduction, multiscale integration, EEG

## Abstract

Conscious experience depends on the coordinated activity of neural processes that span multiple scales: from synapses to whole-brain dynamics. A recently introduced measure, *dynamical independence* (DI), identifies, characterises, and quantifies these multi-scale relationships using an information-theoretic dimensionality reduction approach. Here, we use DI to examine changes in the emergent dynamical organisation in the human brain under three pharmacologically distinct anaesthetic interventions (propofol, xenon, ketamine). Applied to source-reconstructed electroencephalography (EEG), our analysis reveals that propofol and xenon, anaesthetics that abolish conscious report, exhibit more emergent but highly variable dynamic structure, indicating fragmented macroscopic dynamical organisation. Ketamine, which preserves dream-like phenomenology, shows a different pattern relative to wakefulness: reduced overall emergence yet a partial preservation of the macroscopic structure. Further exploratory analyses revealed spatially localised source-level contributions to emergent dynamical structure, highlighting regional variations. Together, our results highlight drug-induced reconfigurations of emergent dynamical structure relative to wakefulness, dissociate the *amount* of emergence from the *organisation* of emergent dynamics, and caution against equating emergence with level of consciousness. Consequently, we suggest that wakeful conscious processing depends not only on integration between neural components within a single scale, but also on integration across scales, broadening currently held assumptions of putative signatures of consciousness.

## 1 Introduction

Transitions between global states of (un)consciousness have often been associated with changes in structure and organisation of the dynamical interactions in neural activity across multiple spatial and temporal scales (Deco et al., 2021; Friston, 2008; Kringelbach et al., 2023; Li et al., 2023; Luppi et al., 2023; Raut et al., 2020). In neuroscience, these spatial scales are broadly and somewhat arbitrarily stratified into single-neuron (microlevel), neural population (mesolevel), and whole-brain (macrolevel) dynamics. Conventionally, it is assumed that smaller units on the lower scale (e.g., neurons or neural populations) coordinate and integrate their activity to generate higher-order patterns that shape global brain dynamics (Breakspear, 2017; Kelso, 2014). Here, higher-order generally refers to coordination of activity, in both the mechanistic and statistical sense, among more than just pairings of two constituent elements.

Efforts to understand how these dynamical interactions relate to consciousness have frequently focused on pairwise couplings, using network-theoretic approaches to examine meso-to-macro transitions and their relevance to cognitive function and consciousness (Avena-Koenigsberger et al., 2018; Bassett & Sporns, 2017; Demertzi et al., 2013; Fornito et al., 2015, 2016; Lee et al., 2019; Rubinov & Sporns, 2010; Uehara et al., 2014). Beyond pairwise interactions, hypergraphs and simplicial complexes capture higher-order *relational* structure at the level of network topology (Battiston & Petri, 2022; Battiston et al., 2020). These frameworks formalise multi-node interactions structurally, whereas the present study instead intends to quantify higher-order statistical dependencies embedded in dynamical time-series data. Relatedly, but with nuanced difference in the method, techniques such as principal component analysis (PCA), brain eigenmodes (Cabral et al., 2022, 2023), and oscillatory harmonic modes (Atasoy et al., 2018; Luppi et al., 2020) have been used to describe large-scale neural dynamics in terms of spatially extended, low-dimensional patterns, offering alternative frameworks to capture meso-scale integration beyond pairwise interactions. While these approaches often presuppose that macroscopic dynamics arise from lower-scale interactions, implicitly invoking the notion of *emergence*, they typically do not set out to measure emergence itself. Moreover, assuming predefined, discretely separable hierarchical scales, they fail to accommodate a *heterarchical* view of the brain’s dynamical and statistical organisation, where interactions can be overlapping and are not strictly contained within any single scale or pair of scales. Addressing this gap, the current study aimed to detect macroscopically organised neural dynamics through their statistical relationships in neurophysiological time-series data, as well as quantify their degree of emergence.

A growing body of theoretical (Seth & Bayne, 2022; Tononi et al., 1998), methodological (Atmanspacher & Bishop, 2007; Barnett & Seth, 2023; Hoel et al., 2013; Rosas et al., 2020), and empirical (Allefeld et al., 2009; Herzog et al., 2022, 2024; Luppi et al., 2022, 2023; Shah et al., 2019; Varley, Pope, Faskowitz, et al., 2023) work supports the notion that aspects of emergent macroscopic interactions appear as putative markers of whole-brain functions and (un)conscious states. Already, information-theoretic measures capturing synergy have revealed that human brains exhibit higher synergy than non-human primate brains (Luppi et al., 2022), and synergy is reduced in patients with disorders of consciousness (Luppi et al., 2023). Synergy, here, is considered a dimension of emergence that quantifies the information about a target variable that is present when considering multiple source variables jointly, and not in any source individually. Another study found that synergistic emergence is greater in human wakefulness than in states associated with diminished consciousness (Luppi et al., 2024). While these measures identify the degree of synergy present in higher-order interactions (interactions of higher dimension than pairings), they do not directly identify the emergent dynamical organisation of these higher-order interactions across spatial scales.

Here, we apply a new approach that defines emergence through *dynamical closure*, whereby the degree of emergence is quantified by assessing how statistically independent macroscopic processes are from their microlevel base. In plain terms, we ask to what extent coarse-grained patterns of activity behave as self-contained dynamical subsystems in their own right.

**Fig. 1.**
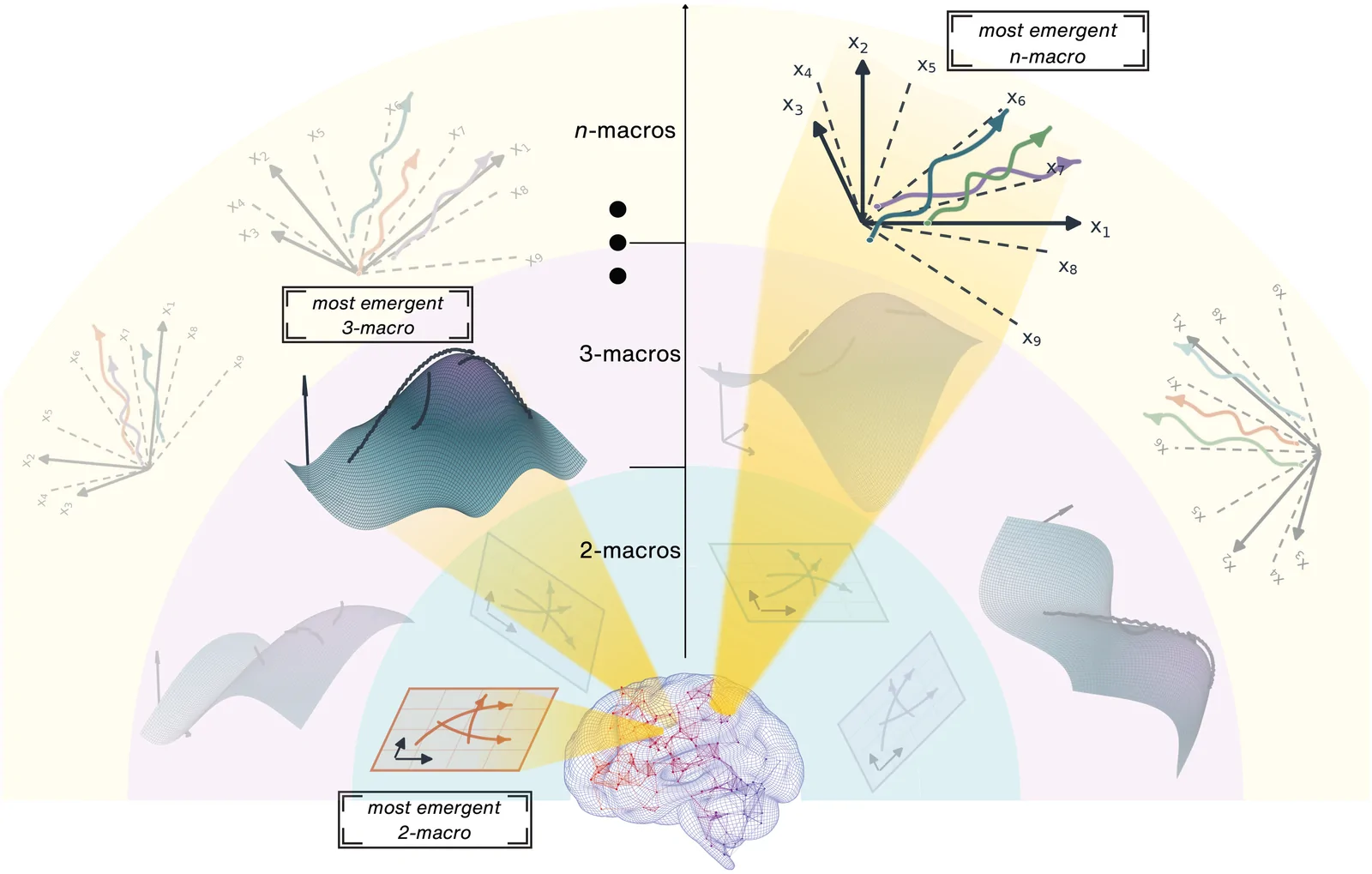
Multiscale emergence framework applied to recorded brain activity. Brain activity is represented across an ordering of spatial scales (vertical axis), where each scale corresponds to a lower-dimensional macroscale subspace (an *n*-macro) onto which the full set of source-level time series are projected. At the base of the ordering, the brain schematic represents the microscale: pairwise interactions between individual brain sources, characterised using network-theoretic or directed statistical measures (e.g., Granger causality). Moving upwards along the scale axis, progressively higher-dimensional macroscopic subspaces are considered, from 2-macros through 3-macros up to *n*-macros. At each scale, four candidate subspaces are depicted, reflecting four independent macros identified across four optimisation runs with minimal dynamical dependence. Each candidate subspace is visualised as a lower-dimensional projection of the full source-level dynamics; that is, a coordinate frame representing how the high-dimensional brain activity unfolds when projected onto that particular subspace. It is important to note that these projected subspaces are distinct from the emergent optimisation landscape over which the search for *n*-macros is conducted; the latter is the landscape being traversed during optimisation and is depicted by the “layer” or scale itself, whereas the former represents the dynamics of brain activity as they appear within each candidate subspace solution. Each stratified hemispheric layer can, therefore, be conceived as an emergent dynamical landscape for that scale, containing multiple solutions. The four candidate subspaces visible at each scale represent four such solutions within that landscape. The most emergent macro at each scale, identified as the candidate with the lowest dynamical dependence *across* optimisation runs, is highlighted by greater opacity and positioned centrally within the illuminated cone. Importantly, the most emergent macro is not necessarily the most stable or dominant solution within the optimisation landscape; it is specifically the solution that *minimises* dynamical dependence, meaning its dynamics are most self-contained with respect to the remaining source-level activity, regardless of the shape or topology of the broader optimisation landscape.

To operationalise this, we employ a technique called dynamical independence (DI; Barnett & Seth, 2023). Crucially, the formalism of DI allows us to quantify how dynamically (in)dependent a macroscopic variable is in informational terms. The converse to DI, dynamical dependence (DD), therefore, provides a quantitative measure of how integrated a system is across scales (levels of description). Furthermore, our method allows us to identify specific low-dimensional subspaces at each scale, and to quantify their DI (and DD) from their lower-level bases. We call these subspaces *n*-macros, where *n* refers to the coarse-graining scale involved, and where the micro-level system from which they emerge is represented by the source variables of the neurophysiological recordings. We provide a full illustrative example in Figure 1.

Formally, DI measures the extent to which knowledge of past micro-states does not significantly improve predictability of a future macro-state beyond the degree to which the macro-state already self-predicts. An *n*-macro is thus deemed emergent to the extent that its future states are largely determined by its own prior states, without reference to prior *micro*-states. In this sense, emergent *n*-macros may be construed as *dynamically self-contained* processes.

Maximising DI across candidate subspaces identifies the *n*-macros that maximise dynamical *in*dependence, and thus are maximally emergent at scale *n*. Functionally, DI, therefore, measures the extent to which large-scale neural patterns realise self-contained dynamical subsystems: processes that evolve according to internally organised dynamics (even if those dynamics *themselves* can be entropic) rather than simply reflecting aggregation of micro-level processes.

In the context of source-reconstructed neurophysiological recordings, the “microscopic” variables represent magnitudes of neural activity at the sources of interest-associated prescribed regional atlas of the brain. We emphasise that, in contrast to functional connectivity analyses, emergent macrovariables in the DI sense should *not* be thought of as “subnetworks” (indeed, *all* system variables may well contribute—to varying degree—to the dynamics of a DI macrovariable at any given scale). Nor do they represent “modes” of the system as a whole: multiple DI macrovariables, at multiple scales, will in general *coexist* as distinct self-contained subprocesses.

Previously, using biophysically plausible neural models, DI was shown to capture the modulation of *emergent dynamical structure* (the arising emergent landscape across all scales) by incrementally varying functional integration and segregation (Milinkovic et al., 2025). To translate this approach to real-world data, here we determine the degree of emergence and we further extend the DI framework by providing a principled method to map the structure of the emergent landscape across all candidate scales, allowing us to move beyond predefined hierarchical levels^1^ and directly characterise how dynamical organisation arises in the conscious states: particularly wake and anaesthesia.

To summarise, it is important to distinguish three related but conceptually distinct notions that are often discussed under the umbrella of “emergence” in neuroscience: *(1)* dynamical closure (as captured by DI (Barnett & Seth, 2023)), *(2)* synergy-based emergence (as captured by partial information decomposition (Mediano et al., 2021) or O-information (Rosas et al., 2019)), and *(3)* signal complexity or diversity (e.g., Lempel-Ziv complexity (M. Schartner et al., 2015)), which reflects compressibility of a signal rather than any notion of “*emergent* complexity”.

Synergy-based approaches quantify the extent to which multiple variables jointly provide irreducible information about a target beyond what is available in any variable individually. In contrast, DI operationalises emergence as dynamical closure: the degree to which a low-dimensional macroscopic process becomes statistically independent of its microscopic base in predicting its own future. Complexity measures, by comparison, quantify signal diversity or compressibility without reference to multiscale organisation. While increased complexity may accompany the emergence *of* structured macroscopic dynamics, complexity itself does not operationalise dynamical closure.

These approaches, therefore, address complementary questions. Synergy asks whether joint interactions carry irreducible information. DI asks whether macroscopic processes become dynamically self-contained relative to their microscopic constituents (scale (in)separability). Complexity measures ask how diverse or unpredictable a signal is. The present work focuses specifically on the second notion. (See Supplementary Table S2 for a comparative summary.)

Anaesthesia-induced behavioural unresponsiveness is often used as a proxy for the loss—or at least—substantial alteration of consciousness (Valli et al., 2023). Different anaesthetic agents produce distinct experiential effects: propofol and xenon typically abolish any reportable conscious experience, whereas ketamine often induces a dissociative, dream-like state (Radek et al., 2023).

These differences in conscious states have been previously linked to changes in the level of cortical and thalamic activity (Eisen et al., 2024, 2026; Kantonen et al., 2023), or to changes in terms of oscillatory activity estimated with the power-spectral density (PSD) (McGuigan et al., 2021; Purdon et al., 2015; Sarasso et al., 2015). More specifically, propofol anaesthesia is associated with a strong increase in delta power also observed in sleep (Murphy et al., 2011) and an increase in frontal alpha (Johnson et al., 2003; Koskinen et al., 2001; Purdon et al., 2013). Comparatively, xenon anaesthesia shifts the distribution towards delta frequencies without alpha power peaks (Johnson et al., 2003). In both, propofol and xenon, evidence suggests that the increase in delta power reflects the occurrence of bistable dynamics marked by alterations between neuronally active (“up”) and neuronally inactive (“down”) states (Breshears et al., 2010; Franks, 2008; Isomura et al., 2006; Massimini et al., 2024). By contrast, ketamine anaesthesia generally leads to a reduction in prominent oscillatory peaks, with alpha power dispersing into theta and high beta bands (Purdon et al., 2013, 2015). Beyond these spectral shifts, anaesthetic-induced unconsciousness involves altered effective connectivity (Hudetz, 2012; Juel et al., 2018), changes in signal diversity (Casali et al., 2013; M. Schartner et al., 2015), variations in the spectral exponent of the PSD (Colombo et al., 2019), and shifts in measures related to criticality (Maschke et al., 2023; Varley et al., 2020).

While these established markers are useful indices of conscious level, they primarily quantify changes in activity, spectral content, or connectivity strength. They do not directly assess whether large-scale patterns of activity form dynamically self-contained emergent subprocesses, nor how such processes reorganise across spatial scales under anaesthesia. In other words, they describe *what* changes, but not whether the system’s *observational* macroscopic organisation becomes more or less (in)separable and (un)structured across scales. Developing tools that explicitly quantify the emergence of macroscopic dynamics, therefore, allows us to move beyond descriptive markers and directly characterise how the brain’s multiscale dynamical organisation is altered across conscious states.

Anaesthetic states thus provide a valuable testing ground for our approach. In the present dataset, we pragmatically classify propofol and xenon conditions as “no conscious-report” states, since subjects reported no conscious experiences upon regaining responsiveness, whereas ketamine conditions are classified as “affirmed conscious-report”, based on the post-hoc dream-like reports provided by participants. We emphasise that these behavioural categories serve as proxies for reported experience rather than direct measures of conscious level per se.

By applying DI to open-source EEG data recorded during these conditions (Sarasso et al., 2015), we examined how emergent dynamical organisation differs across pharmacologically distinct states that vary in reportability and phenomenology. Our aim is, therefore, not to infer conscious level directly from DI, but to determine whether drug-induced alterations in emergent structure can be detected, and whether these changes align with established distinctions between unreportable (propofol, xenon) and altered but reportable (ketamine) conditions, where reportability is taken as a proxy for conscious level. In particular, we test whether DI can distinguish wakefulness from propofol- and xenon-induced conditions, as well as differentiate wake from ketamine, which is associated with preserved yet altered conscious experience.

## 2 Methods

### 2.1 Data overview

The present analysis solely utilised the spontaneous 61-channel EEG data of the open-source recordings described in Sarasso et al. (2015). Full details on participant selection, anaesthetic dosing, clinical monitoring, and retrospective assessments of participants’ subjective experience can be found in the Supplementary Material of that original work. Here, we offer a brief summary.

#### 2.1.1 Participants

A total of 14 healthy adults (7 females), aged between 18 and 28 years, provided written informed consent and participated in the study, which was approved by the local ethics committee of the University of Liège, Belgium. All participants underwent medical and physical screening to exclude conditions that might interfere with anaesthetic administration. They were then assigned to one of three anaesthetic protocols: propofol (*n* = 4), xenon (*n* = 5), or ketamine (*n* = 5). Although the original dataset included transcranial magnetic stimulation (TMS) sessions, only the spontaneous EEG recordings were analysed in the present study (Sarasso et al., 2015).

#### 2.1.2 Experimental procedures

All testing was conducted at the Centre Hospitalier Universitaire (CHU) in Liège, with each participant receiving one anaesthetic agent only (Sarasso et al., 2015). A standard dose was administered to achieve unresponsiveness (Ramsay Scale 6), followed by continuous spontaneous EEG recording. A 10-minute pre-anaesthetic wake segment was recorded as baseline. Anaesthesia was then ceased, and participants were monitored until full responsiveness.

Vital signs (blood pressure, oxygen saturation, exhaled ${\text{CO}}_{2}$, axillary temperature) were tracked throughout. Metoclopramide was administered to reduce nausea risk. After recovery, retrospective reports of any conscious experience during anaesthetic unresponsiveness were collected.

#### 2.1.3 Conscious reports

Participants under propofol and xenon reported no conscious experience, except one xenon case with a vague non-specific sensation. In contrast, ketamine participants reported dream-like experiences, categorised here as *affirmed conscious-report* versus *no conscious-report*.

An example after ketamine admission (abridged from Sarasso et al., 2015):

“I felt myself falling backwards into space, then in a futuristic vessel discussing an abstract problem with acquaintances. We disagreed, and I concluded all my prior beliefs were wrong. The scene shifted to a slippery space with a giant white sphere, then to a machine-filled room where I sought a solution using a technology that did not yet exist. Finally, the space flattened into a bright white form filling my vision: beautiful, impressive, and strange, yet without anxiety.”

### 2.2 Preprocessing and analysis of EEG data

Spontaneous EEG (61 channels, 1,450 Hz) was high-pass filtered at 1 Hz, low-pass filtered at 250 Hz, notch filtered at 50/100 Hz, re-referenced to the global average, and downsampled to 256 Hz. Artefact rejection was performed in MNE-Python via projection of artefact vectors and average referencing. Continuous signals were segmented into non-overlapping 2-second epochs, aligned with anaesthetic stages, and treated as stationary realisations from an underlying stochastic process (Barnett & Seth, 2014; Barnett et al., 2009). Usable epoch counts per participant and condition are provided in Supplementary Table S1.

### 2.3 Source reconstruction

Noise covariance was estimated from cleaned continuous EEG. Source analysis used the fsaverage template to ensure comparability, with a mid-resolution cortical mesh (ico4) and three-layer BEM model in MNE-Python. Sensor locations were registered to the template head, and a forward solution was computed with 5 mm source–skull minimum distance, producing the leadfield matrix.

Cortical activity was estimated per epoch using an LCMV beamformer (make_lcmv, apply_lcmv_epochs), with 5% regularisation, weight normalisation (unit-noise gain), NAI depth compensation, and max-power orientation mode. Resulting source time series were parcellated into 46 anatomical regions using the reduced HCP atlas (Supplementary Fig. S1), extracted in mean_flip mode. Regions with zero-valued output were excluded. Final data were structured as epochs × time × regions and saved in both .csv and .mat formats for dynamical independence analysis.

All preprocessing, including Z-score normalisation, was performed per participant and condition, except for PSD analysis (see Supplementary Material). Wake data were averaged within, but not pooled across, anaesthetic groups.

Source reconstruction from the 61-channel EEG is inherently limited in spatial precision and subject to the ill-posed nature of the inverse problem in source reconstruction methods. This concern is somewhat lessened since the LCMV beamformer we used reduces spatial leakage significantly, relative to minimum-norm approaches (Van Veen et al., 1997). However, residual source spread and depth bias may remain, motivating caution in interpretation. Spatial leakage can artefactually increase correlations between neighbouring regions, potentially influencing multivariate interaction measures capturing directed information flow between regions (Mahjoory et al., 2017). However, in the present analysis, reconstructed sources were parcellated into 46 anatomically defined regions, reducing fine-grained voxel-level leakage where such effects are typically strongest. All model fitting and condition contrasts were computed separately for each participant, meaning that statistical comparisons were based on within-subject differences before group-level aggregation, such that any systematic reconstruction bias would be consistent across conditions. Furthermore, our spatial localisation method does not inherit the bias of connectivity-based, directed information flow metrics as it is based on source contribution to macros, rather than information flow between sources. Nonetheless, we interpret spatial contribution findings cautiously and exploratively and focus primarily on multiscale dynamical organisation rather than on precise anatomical claims.

### 2.4 Vector autoregressive and state-space modelling

To investigate emergent dynamical structure in the EEG data, Vector Autoregressive (VAR) models were fitted to the source-level signals using the MVGC2 toolbox (Barnett & Seth, 2014, 2015). Model fitting was performed separately for each subject and condition. For each dataset, the optimal model order was selected by evaluating Akaike (AIC) and Bayesian (BIC) information criteria over a candidate range up to 30 lags. Given BIC’s tendency to underfit in practice, we selected AIC as our primary criterion, which typically resulted in model orders between 8 and 20.

VAR coefficients and noise covariances were then estimated via least-squares regression using MVGC2’s tsdata_to_var function, yielding a model of the form:

$${\text{X}}_{t}=\sum_{k=1}^{p}A^{\left(k\right)}{\text{X}}_{t-k}+\varepsilon_{t},$$ (1)

where ${\text{X}}_{t}$ is the *N*-dimensional multivariate source time series, *A*^(^*^k^*^)^ the autoregressive coefficient matrix at lag *k*, and $\epsilon_{t}$ a zero-mean white noise (iid and serially uncorrelated) residual process with covariance matrix *V*.

To facilitate further analysis, each VAR model was converted into an equivalent state-space (SS) model in innovations form (Hannan & Deistler, 2012). This was done using MVGC2’s var_to_ss routine, which constructs the companion-form state transition matrix *A_ss_*, observation matrix *C*, Kalman gain matrix *K*, and innovation noise covariance *V_ss_* (in our case, since ε*_t_* is the same residual process as the VAR model, this is equivalent to *V*). The system is described by the innovations state-space equations:

$${\text{Z}}_{t+1}=A_{ss}{\text{Z}}_{t}+K\varepsilon_{t}$$ (2a)

$${\text{X}}_{t}=C{\text{Z}}_{t}+\varepsilon_{t},$$ (2b)

where ${\text{Z}}_{t}$ is the latent state vector of dimension *r*, which dynamically generates the observed activity ${\text{X}}_{t}$ through linear mapping and innovation-driven temporal evolution.

Finally, spectral properties of the fitted systems were evaluated by transforming the VAR coefficients into a frequency-domain transfer function *H*(ω) using var2trfun, allowing for computationally efficient calculation of dynamical dependence.

While not central to our main analysis, pairwise-conditional Granger-causal estimates were computed without a statistical threshold and provided in the Supplementary Texts S2 and S3, and illustrated in Supplementary Figure S3.

### 2.5 Dynamical independence: Information-theoretic dimensionality reduction

After estimating state-space parameters, we evaluated dynamical dependence (DD) to identify emergent macroscopic variables in the temporal domain. Dynamical independence (DI), an information-theoretic dimensionality-reduction technique (Barnett & Seth, 2023), is operationalised here in terms of linear modelling, identifying maximally-DI macrovariables then involves optimisation on a matrix manifold to minimise DD. Unlike PCA (Bishop & Nasrabadi, 2006; Pearson, 1901) or Maximum Autocorrelation Factors (MAF) (Churchland et al., 2012; Switzer, 1984), DI is sensitive to the full temporal dependency structure underlying emergent dynamics.

A coarse graining in this context is a linear map $M:{\mathbb{R}}^{N}\to {\mathbb{R}}^{n}$ projecting high-dimensional source data $\text{x}\in {\mathbb{R}}^{N}$ to $\text{y}=M\text{x}\in {\mathbb{R}}^{n}$. DD is quantified with the time-domain Granger causality statistic as

$$F_{\text{X}\to M\text{X}}=\frac{1}{2\pi }\int_{-\pi }^{\pi }f_{\text{X}\to M\text{X}}\left(\omega \right)d\omega ,$$ (3)

$$f_{\text{X}\to M\text{X}}\left(\omega \right)=\text{log}\left|MS\left(\omega \right)M^{T}\right|,$$ (4)

where *S*(ω) is the cross-power spectral density (CPSD) matrix computed from the model transfer function *H*(ω) and innovations covariance *V* as $S\left(\omega \right)=H\left(\omega \right)VH{\left(\omega \right)}^{\text{*}}$. We remark that $F_{\text{X}\to M\text{X}}$ thus defined is the time-domain Granger causality, with an information-theoretic interpretation as *transfer entropy* (Barnett & Bossomaier, 2012; Barnett et al., 2009), from the microvariable X to the macrovariable MX (Barnett & Seth, 2023), and as such is invariant under non-singular transformations of both source space ℝ*^N^* and target space ℝ*^n^*, so without loss of generality we may restrict *M* to orthonormal *n*×*N* matrices on the Stiefel manifold $V_{N}\left(n\right)$, parametrising points on the Grassmannian $G_{N}\left(n\right)$.^2^

**Definition 1 (*****n*****-macro)**. *An n*-*macro is an n-dimensional (linear)*^3^ *subspace of* ℝ*^N^*; *it is* emergent *if it locally minimises DD* on $G_{N}\left(n\right)$.

Optimisation proceeds in two stages: (i) minimise the proxy

$$F^{*}\left(\text{X}\to M\text{X}\right)=\sum_{k=0}^{r-1}{\left\|MQ_{k}M^{T}\right\|}^{2},\;Q_{k}=CA^{k}K,$$ (5)

over the space of *n*-macros $M\in V_{N}\left(n\right)$, where (C,A,K) are state-space parameters, then (ii) refine via (3). The *Q_k_* are pre-computed, making stage (i) computationally efficient.

It offers the following advantages over other dimensionality-reduction techniques such as PCA/MAF:

- **Scale flexibility:** Identifies macroscopic variables at any scale 1≤n<N and, unlike PCA, quantifies emergence via DD to find subspaces that are both low-dimensional and maximally dynamically independent from their microscopic substrates.
- **Sensitivity to dynamics:** Incorporates the full temporal structure, including multi-lag dependencies, whereas PCA captures only contemporaneous variance and MAF maximises single-lag autocorrelation.
- **Information-theoretic basis:** Implemented via Granger causality, allowing DD to be interpreted as information flow from the microscopic system to the macro, providing a principled emergence measure.
- **Source-level contributions:** The contribution of individual sources to each *n*-macro may be naturally quantified, enabling spatial localisation of macroscopic dynamics (see Methods Section 2.5).

#### 2.5.1 Characterising the emergent dynamical structure

For each subject and condition, 100 random initialisations on $V_{N}\left(n\right)$ were optimised for 2≤n≤9 using gradient descent (up to 10^4^ iterations, tolerance $<10^{-10}$ ). Pre-optimisation with F^*^ was followed by refinement with *F*, integrating over [0, *π*].

To assess the emergent dynamical landscape, we computed all pairwise *principal angles *θ*_j_* between optimised *n*-macros, yielding 100 × 100 similarity matrices. For equal-dimension subspaces *M*_1_, *M*_2_, the canonical Grassmannian metric is

$$d\left(M_{1},M_{2}\right)=\sqrt{\sum_{j=1}^{n}\theta_{j}^{2}},\theta_{j}\in \left[0,\pi /2\right],$$ (6)

normalised to [0, 1] via ${\left(\pi /2\right)}^{-1}$ . Small distances indicate a stable, consistently identified dynamical structure; large, variable distances indicate fragmented or heterogeneous organisational structure.

#### 2.5.2 Characterising source-level contributions to n-macros

To localise contributions of individual sources to each emergent *n*-macro, we computed the angle between each source coordinate axis in the *N* = 46-dimensional space and the *n*-macro subspace. The squared cosine of this angle quantifies the *regional embedding*, that is, the spatial localisation of the macroscopic variable.

Under the null hypothesis that an *n*-macro is drawn uniformly at random from the Grassmannian $G_{N}\left(n\right)$, the squared cosine of the angle between a fixed unit vector (source) and the subspace is distributed as Frankl and Maehara (1990):

$${\text{cos}}^{2}\left(\theta \right)\sim \text{Beta}\left(\frac{n}{2},\frac{N-n}{2}\right).$$ (7)

Comparing observed values to this Beta distribution yields a probability-theoretic test for whether a source contributes more (or less) than expected by chance. This standardised effect size provides a principled measure of the *structural contribution* of each region to the emergent macroscopic process.

### 2.6 Statistical comparison of dynamical dependence values

Dynamical dependence (DD) values between wakefulness and anaesthetic states were compared across macroscales using a groupwise (participant-stratified) Mann–Whitney U (MWU) test (two-tailed, non-parametric). For each participant *i* and scale *n*, we compared the distributions of DD values obtained across optimisation runs in wake (*n*_1_ runs) and anaesthesia (*n*_2_ runs). This yields a within-subject Mann–Whitney statistic *U_i_*, which was converted to a Z-score under the asymptotic normal approximation:

$$z_{i}=\frac{U_{i}-\mu_{U}}{\sigma_{U}},\;\mu_{U}=\frac{n_{1}n_{2}}{2},\;\sigma_{U}=\sqrt{\frac{n_{1}n_{2}\left(n_{1}+n_{2}+1\right)}{12}}.$$ (8)

Group-level effects (per anaesthetic type) were obtained by aggregating the participant-wise Z-scores, assuming independence across participants. This corresponds to a Stouffer-equivalent combination of subject-level test statistics:

$$Z_{\text{group}}=\frac{1}{\sqrt{N}}\sum_{i=1}^{N}z_{i},p_{\text{group}}=\frac{1}{2}\text{erfc}\left(\frac{Z_{\text{group}}}{\sqrt{2}}\right).$$ (9)

Thus, wake versus anaesthetic contrasts were performed within subject (across optimisation runs), and group-level inference was obtained by aggregating subject-specific statistics rather than pooling observations across subjects. Within each participant, the wake and anaesthetic samples entering the MWU are the independently initialised optimisation runs, which are mutually independent by construction, satisfying the independence assumption of the test despite the within-subject design. Results were controlled for multiple macroscales and conditions using a Benjamini–Hochberg FDR correction at significance α=0.05 .

These condition-level inferences are based on direct statistical contrasts (e.g., wake vs propofol, wake vs xenon, wake vs ketamine) rather than on comparisons of significance levels across separate tests. As a supplementary analysis, we tested whether DD condition effects differed between anaesthetic agents. At each macroscale (2≤n≤9 ), we ran mixed ANOVAs with condition (Wake vs Anaesthesia) as the within-subject factor and agent as the between-subject factor, first across all three agents and then as planned pairwise 2 × 2 contrasts. P-values were FDR corrected across macroscales within each interaction family.

### 2.7 Statistical comparison of similarity-matrix values

For each subject, condition, and scale (*n* = 2–9), we computed similarity matrices (100 × 100) from optimised *n*-macros, extracted the upper triangle, and calculated the Structure–Fragmentation (SF) index: proportion of values $<10^{-4}$ . SF indices were analysed via rmANOVA (fixed effects: macro, condition; random effect: subject), with partial η^2^ reported. Post hoc paired Wilcoxon tests were FDR corrected. We ran the same supplementary analysis as above to explore the between-subject anaesthetic differences in SF values.

### 2.8 Statistical comparison of β-values

Regional contributions to each *n*-macro were quantified using the hyperplane axis–angle β statistic (squared cosine of alignment between a source vector and the macro subspace). Under the null hypothesis of random orientation on the Grassmann manifold, as already mentioned above, a β statistic follows a Beta distribution

$$\beta \sim \text{Beta}\left(\frac{m}{2},\frac{n-m}{2}\right),$$

where *m* denotes the macrodimensionality and *n* the dimensionality of the source space. For each subject, condition, and macroscale, region-wise significance was evaluated against this analytical null distribution using two-tailed tests with Bonferroni correction across regions.

To compare wakefulness and anaesthetic conditions at the group level, we employed a grouped (participant-stratified) Mann–Whitney U (MWU) test separately for each region. Within each subject, vectors of β-values across macroscales were compared between wake and anaesthesia, yielding subject-specific U statistics. These were aggregated across subjects assuming independence between participants, converted to asymptotic Z-scores, and evaluated using two-tailed tests. Dependency-robust false discovery rate correction (FDRD) was applied across the 46 regions.

The grouped (participant-stratified) formulation of the MWU specifically addresses the within-subject nature of the design: rather than pooling observations across participants, which would confound subject identity with condition, a U statistic is computed within each participant by comparing their independent wake and anaesthetic optimisation runs, and these are subsequently aggregated at the group level. Because each optimisation run constitutes an independent random initialisation on the Grassmann manifold, the two samples entering each within-subject U test are independent, satisfying the assumptions of the MW procedure.

Thus, wake versus anaesthetic contrasts for β-values were performed within subject and aggregated across participants without pooling observations across subjects.

### 2.9 Scope of statistical inference

Primary statistical inference focused on within-subject comparisons between wakefulness and the anaesthesia condition measured in the same participant. This paired structure was used for the main DD and SF analyses, allowing each participant to serve as their own wakefulness baseline. Participants were assigned to a single anaesthetic condition only (propofol: *n* = 4; xenon: *n* = 5; ketamine: *n* = 5); thus, anaesthetic agent was a between-subject factor rather than a within-subject manipulation. Accordingly, wake-versus-anaesthesia effects can be interpreted as paired within-agent condition effects, whereas between-agent comparisons necessarily test whether these paired condition effects differ across independent participant groups.

As a supplementary analysis, we test whether the magnitude of the wake-to-anaesthesia effect differed between anaesthetic agents by performing additional scale-resolved mixed ANOVA interaction analyses. At each macroscale (2≤n≤9 ), we first ran a three-agent mixed ANOVA with condition (Wake vs Anaesthesia) as a within-subject factor and anaesthetic agent (propofol, xenon, ketamine) as a between-subject factor. The condition × agent term tests whether the anaesthesia-induced change differs anywhere among the three agents. We then ran planned pairwise 2 × 2 mixed ANOVAs for each drug pair at each macroscale, again with condition as the within-subject factor and agent as the between-subject factor. In these pairwise models, the condition × agent term directly tests whether the wake-to-anaesthesia effect differs between two anaesthetic agents.

These between-agent interaction analyses should be interpreted cautiously because anaesthetic agent was not manipulated within subject and group sizes were modest. They are, therefore, used to test whether the observed paired wake-to-anaesthesia effects differ across independent agent groups, not to infer within-subject pharmacological specificity. The principal statistical claims of the study remain the within-agent wake-versus-anaesthesia effects, with between-agent interaction tests reported as scale-resolved secondary analyses.

## 3 Results

To summarise, we analysed spontaneous EEG recordings from 14 healthy adults (7 females, aged 18–28 years ), each of whom underwent a single anaesthetic protocol involving either propofol (*n* = 4), xenon (*n* = 5), or ketamine (*n* = 5). Prior to anaesthesia, all participants completed a baseline wakefulness recording session, lasting 10 minutes, which served as the comparator condition for subsequent analyses (see Supplementary Fig. S2 for power-spectral density analysis). These wakeful recordings were collected immediately prior to the administration of anaesthetic, within the same session, ensuring within-subject comparisons across conditions. Anaesthetic depth was titrated to unresponsiveness (Ramsay Scale 6), and EEG was continuously recorded throughout. Only the spontaneous EEG segments before (wakefulness), and during, unresponsive anaesthesia were used in the present analysis. As previously reported (Sarasso et al., 2015), only participants in the ketamine group retrospectively affirmed any conscious experiences upon emergence; participants in the propofol and xenon groups reported no such experiences.

In the subsequent analysis, we first quantified the *degree of emergence* by identifying lower-dimensional patterns of source-reconstructed EEG activity (*n*-macros) that evolve as independently as possible from their underlying microscopic sources. Operationally, this corresponds to minimising DD. Lower DD, therefore, indicates that a large-scale pattern behaves like a dynamically self-contained process. Second, to characterise the *emergent dynamical structure*, we examined whether particular *n*-macros consistently reappeared across independent optimisation runs. The recurrence of similar low-dimensional subspaces suggests the presence of stable, dominant higher-order processes within a given pharmacological condition. In this way, we move beyond quantifying how much emergence is present, to identifying which macroscopic dynamical patterns reliably organise brain activity in each state.

### 3.1 The dynamical dependence of macroscopic variables across higher-order scales

To measure the degree of emergence, we quantified the DD of *n*-macros with respect to the underlying microlevel source processes across multiple spatial scales. We treat the time-series of the 46 source-reconstructed EEG signals as the microscopic level from which macroscopic dynamics emerge. In this formulation, DD quantifies the extent to which residual predictive influence from the source level persists in shaping the future evolution of a macroscopic process.

*Emergent n*-macros are defined as those lower-dimensional subspaces of the source activity that *minimise* DD, identified through repeated optimisations (*N* = 100 runs; see Methods Section 2.5.1). Due to the non-convex nature of the optimisation landscape, different runs may converge on distinct local minima, indicating the presence of multiple near-optimal macroscopic solutions at each scale.

**Fig. 2:**
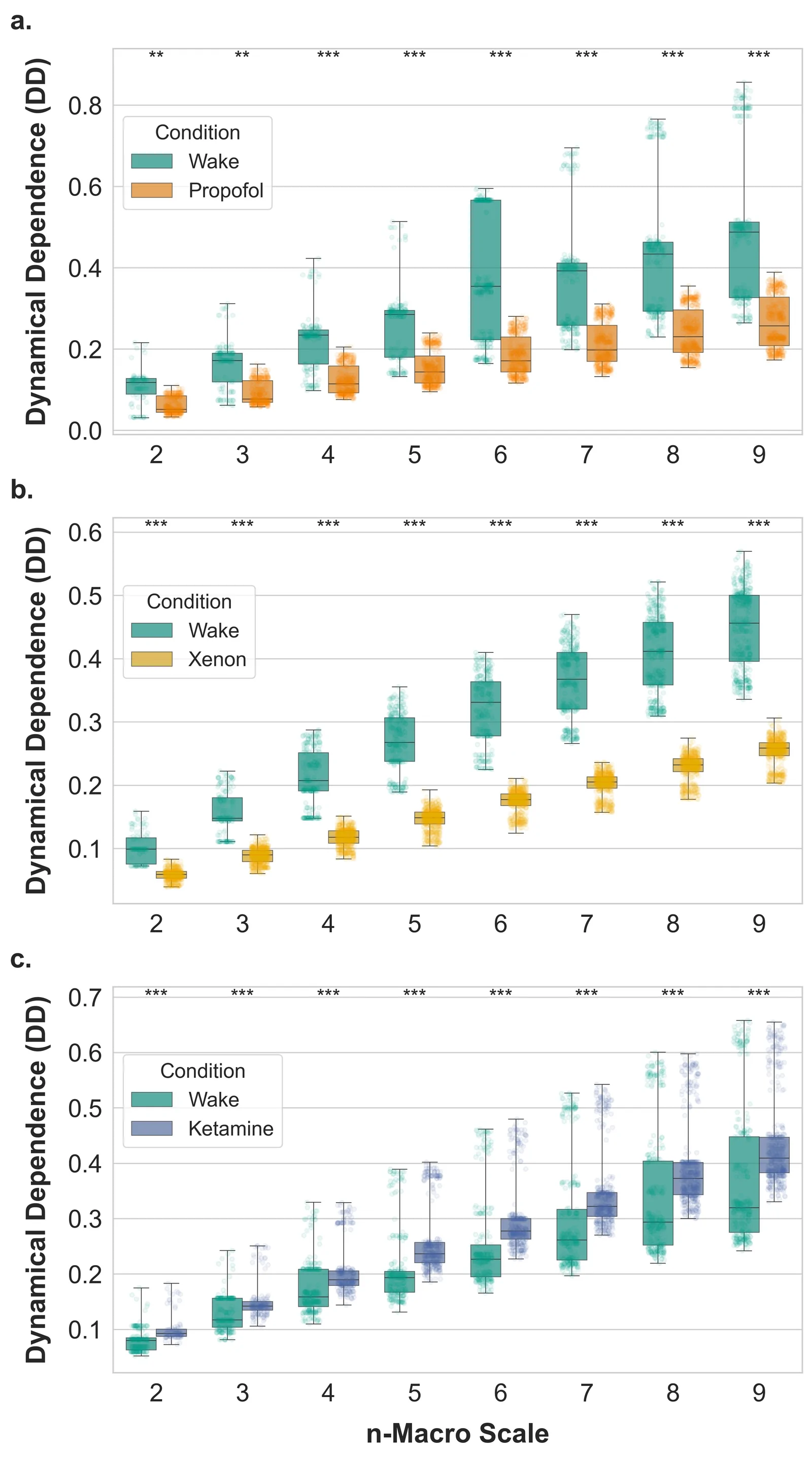
Dynamical dependence across global states of consciousness and macroscales. The figure shows the distribution of dynamical dependence (DD) values across different macroscales (2≤n≤9 ) for three anaesthetic conditions compared with wakefulness. (a) Wake versus propofol, (b) Wake versus xenon, and (c) Wake versus ketamine. For each macroscale, box plots display the median (central line), interquartile range (box), and full range (whiskers) of DD values. Individual data points are shown as semi-transparent dots, with teal representing wake measurements and condition-specific colours for each anaesthetic (orange for propofol, yellow for xenon, and lilac for ketamine). Statistical significance between wake and anaesthetic conditions is indicated by asterisks above each macroscale (**: p<0.001 , ***: p<0.0001 ). Higher DD values indicate stronger dynamical coupling—lower emergence—between higher-order *n*-macros and the lower-level sources, while lower values suggest increased emergence. The consistent differences across macroscales demonstrate robust changes in the degree of emergence of macroscopic brain dynamics under different states of consciousness.

This procedure yields 100 candidate *n*-macros per spatial scale (2≤n≤9 ) for each participant (*N* = 14). For each scale and condition, we, therefore, obtained distributions of minimised DD values across optimisation runs, allowing comparisons between wakefulness (W) and anaesthetic conditions (propofol, xenon, ketamine; P, X, K, respectively). In Figure 2 we compare the control condition (teal for wakefulness) and anaesthesia (orange for propofol, yellow for xenon, lilac for ketamine).

To determine whether the degree of emergence differed significantly across conditions and spatial scales, we applied non-parametric comparisons of DD distributions at the individual participant level. These participant-specific comparisons were then combined across groups using a Z-score aggregation procedure (see Methods Section 2.6), allowing us to test whether DD values under anaesthesia were systematically shifted relative to wakefulness in terms of stochastic dominance (Barrett & Donald, 2003). In practical terms, this tests whether one condition consistently exhibited lower (or higher) dynamical dependence than another across optimisation runs. This approach accounts for inter-subject variability and is statistically equivalent to Stouffer’s method (Whitlock, 2005) for combining independent z-values. Group-level significance was evaluated using false discovery rate (FDR) correction across macroscales.

The results reveal a drug-induced modulation of emergence, with each anaesthetic condition producing a change in pattern relative to wakefulness. This is operationalised as changes in the degree to which observational macroscopic dynamics remain predictively influenced by underlying source-level EEG processes. As shown in Figure 2a, compared with wakefulness, propofol yields significantly lower DD values across all examined spatial scales. Lower DD indicates reduced source-level influence on the macrodynamics corresponding to increased dynamical independence (i.e., strong dynamical closure). Under propofol, therefore, the identified macroscopic processes are more independent with respect to their source-level processes.

Xenon also exhibits significantly reduced DD values relative to wakefulness at all higher-order scales (Fig. 2b), indicating increased dynamical independence of the identified macros under xenon.

Ketamine produces a qualitatively different pattern relative to wakefulness: DD values across all *n*-macros are significantly higher than in wakefulness (Fig. 2c). Higher DD reflects stronger predictive influence from the source-level activity onto the observed macroscopic processes, implying reduced dynamical closure at the macrolevel. Comparatively to wake, under ketamine, higher-order dynamics remain more tightly coupled to their underlying source-level processes.

We next tested whether the DD condition effect differed between anaesthetic agents. For each macroscale, we first ran a three-agent mixed ANOVA with condition (Wake vs Anaesthesia) as the within-subject factor and anaesthetic agent (propofol, xenon, ketamine) as the between-subject factor. This model tests whether the anaesthesia-induced DD change differs anywhere among the three agents. The three-agent condition × agent interaction did not survive FDR correction at any macroscale (F(2,11)=2.72 –3.21, p=.080 –.110, q=.110 , $\eta_{p}^{2}=.331$ –.368; Supplementary Table S3).

We then performed pairwise 2 × 2 mixed ANOVAs for each drug pair and macroscale, again with condition as the within-subject factor and agent as the between-subject factor. These pairwise condition × agent interactions test whether the DD condition effect differs between two specific agents. Propofol and xenon did not differ in their DD condition effects at any scale (F(1,7)=0.00 –0.02, p=.893 –.997, q=.997 , $\eta_{p}^{2}=.000$ –.003; Supplementary Table S4). Propofol and ketamine also did not differ significantly at any scale (F(1,7)=2.55 –3.13, p=.120 –.154, q=.154 , $\eta_{p}^{2}=.267$ –.309; Supplementary Table S4). By contrast, xenon and ketamine differed significantly in their DD condition effects at every macroscale (2≤n≤9 ; F(1,8)=7.11 –8.78, p=.018 –.028, q=.028 , $\eta_{p}^{2}=.471$ –.523; Supplementary Table S4), with xenon showing reduced DD change and ketamine showing increased DD change.

Together, these results indicate that anaesthesia alters the dynamical dependence of emergent macroscopic variables, but not in a uniform direction across all agents. Propofol and xenon each reduced DD relative to wakefulness, consistent with greater dynamical independence of the identified macros and reduced residual source-level influence. Ketamine, by contrast, increased DD. The direct interaction analyses support a dissociation between the anaesthetic action of xenon and ketamine on the DD values, whereby differences are significant at every macroscale, whereas propofol did not differ significantly from either xenon or ketamine.

### 3.2 Revealing dynamical structure of *n*-macros across conditions

To characterise emergent dynamical organisation across higher-order spatial scales, we examined whether optimisation runs within each participant tended to converge on similar macroscopic solutions at a given scale, *n*. Specifically, we assessed the extent to which distinct optimisations yielded convergent *n*-macros, quantified via pairwise subspace angles between resulting solutions (see Methods Section 2.5.1).

**Fig. 3:**
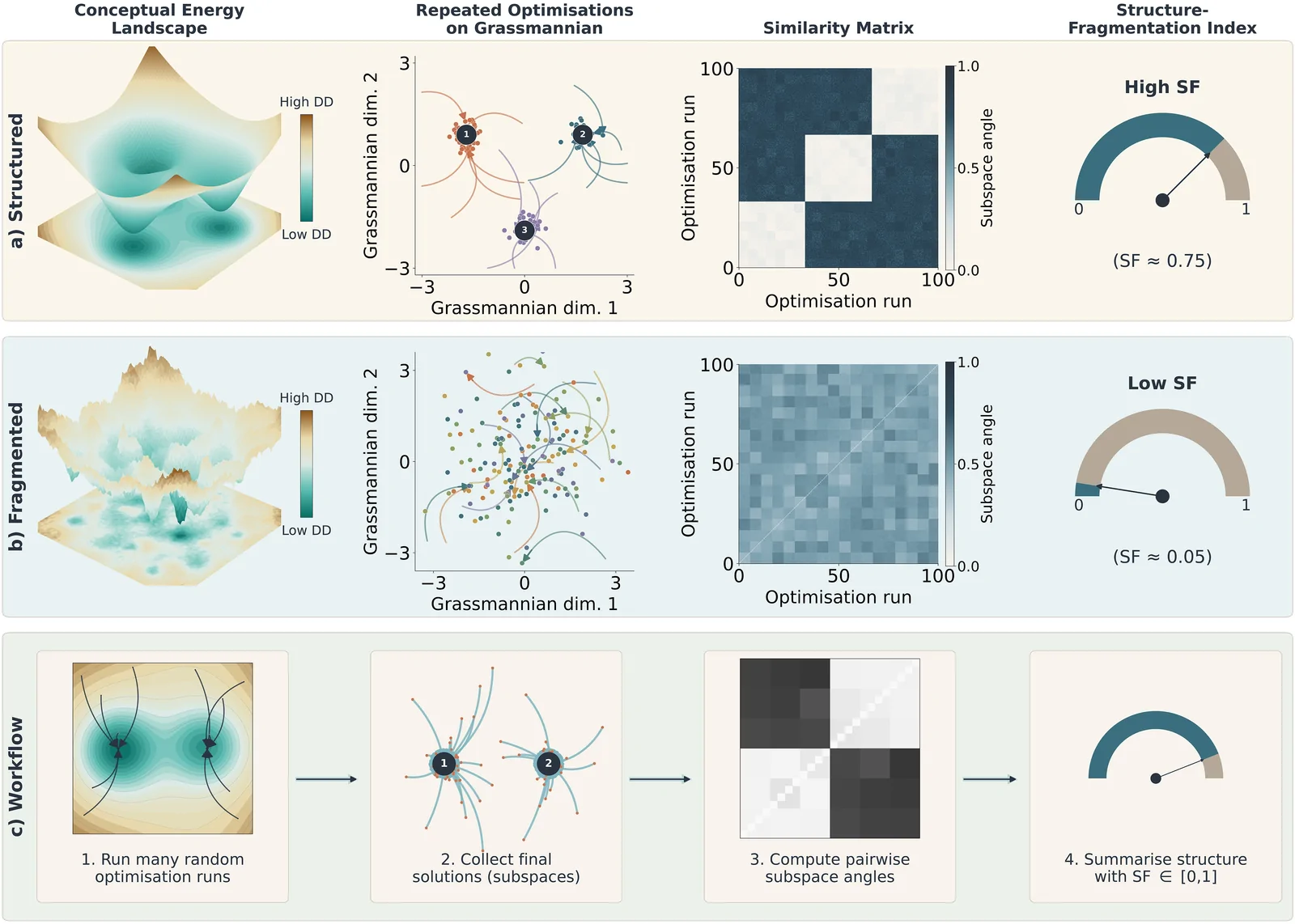
Structure–Fragmentation overview across optimisation regimes. (a) Structured regime: a conceptual energy landscape with distinct basins (DD, depth-derived displacement; lower DD in deeper valleys), repeated optimisations on Grassmannian G(k,p) cluster into consistent solutions, the similarity matrix shows block structure (low within-basin subspace angles), and the resulting Structure–Fragmentation index (SF) is high. (b) Fragmented regime: a rough/noisy landscape with many irregular minima, dispersed Grassmannian solutions, and a coarse-grained similarity matrix without stable block structure, yielding low SF. (c) Workflow summary: random initialisations, collection of converged subspaces, pairwise angle-based similarity estimation, and final SF summarisation. High SF dominant, reproducible solution structure; low SF indicates diffuse, unstable structure across runs.

For instance, two optimisation runs may produce *n*-macros *M*_1_ and *M*_2_, respectively. These may be nearly identical or substantially distinct. We quantified their similarity using the normalised principal subspace angle between *M*_1_ and *M*_2_, where values approaching zero indicate near-identical macroscopic subspaces and larger angles reflect increasingly distinct solutions. To aid interpretation of the similarity matrices and the Structure–Fragmentation (SF) index that follows, we first illustrate an example of structured versus fragmented emergent optimisation landscapes (Fig. 3).

We interpret a proliferation of distinct solutions as reflecting a *fragmentation* of the emergence dynamical landscape, operationally defined as the absence of a single dominant or repeatedly recovered macroscopic process at a given scale.

The resulting pairwise similarity matrices, derived from 100 optimisation runs per subject and ordered by ascending DD, revealed that under wakefulness, many runs converged on identical or near-identical *n*-macros, forming clear blocks of high similarity. This pattern was most evident at lower-to-intermediate spatial scales but persisted, albeit with some dispersion, at higher scales.

**Fig. 4:**
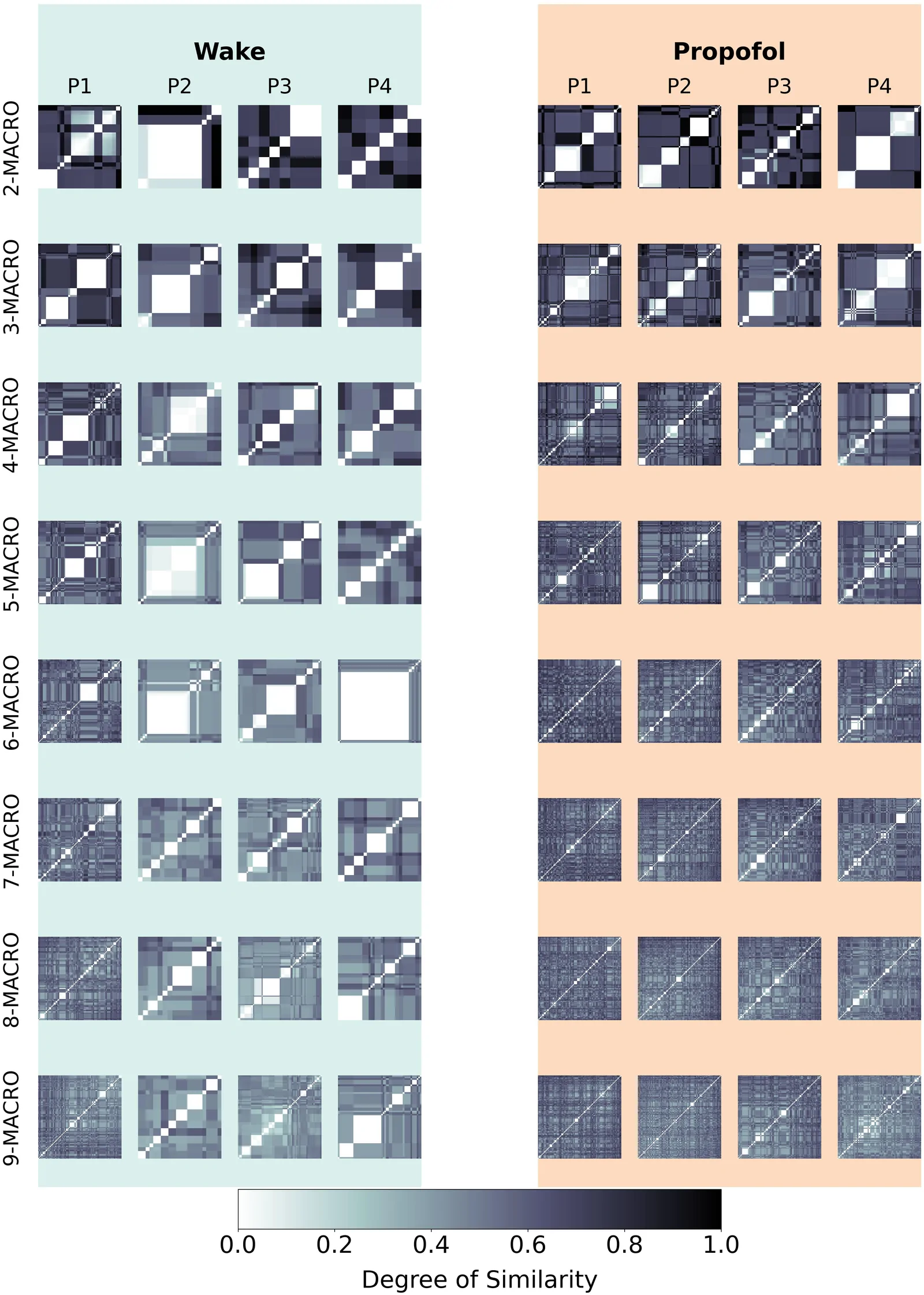
Propofol-induced changes in emergent dynamical structure on higher-order spatial scales. Similarity matrices displaying the pairwise distances (subspace angles) between *n*-macros across optimisation during Wake (left) and Propofol (right) conditions. The colour scale represents the principal angles (see Methods Section 2.5.1) between *n*-macros where greater dissimilarity between *n*-macros *M*_1_ and *M*_2_ is indicated with darker colours (black = orthogonal), and lighter colours signify greater similarity (white = identical). Both the *x*-axis and *y*-axis of the matrix correspond to the number of optimisation runs.

In contrast to wake, under propofol (Fig. 4), this convergence deteriorated markedly. The similarity matrices showed greater dispersion, reflecting increased heterogeneity and fragmentation in the emergent solutions. Importantly, this fragmentation does not imply increased DD (which was reduced under propofol), but rather reduced stability and reproducibility of the identified macros across optimisation runs.

This pattern is indicative of flatter optimisation landscape containing multiple near-optimal minima (low DD *n*-macros), none of which form a strongly dominant basin of attraction.

Importantly, fragmentation should not be interpreted as the system transitioning between mutually exclusive macroscopic processes over time. Rather, it reflects the presence of multiple comparably independent macroscopic subspaces that can be identified from the same data, suggesting reduced dominance at any single dynamical process on the candidate scale. This proliferation of distinct, yet similarly independent macroscopic processes provides a quantitative characterisation of the fragmentation of emergent dynamical structure under anaesthesia.

**Fig. 5:**
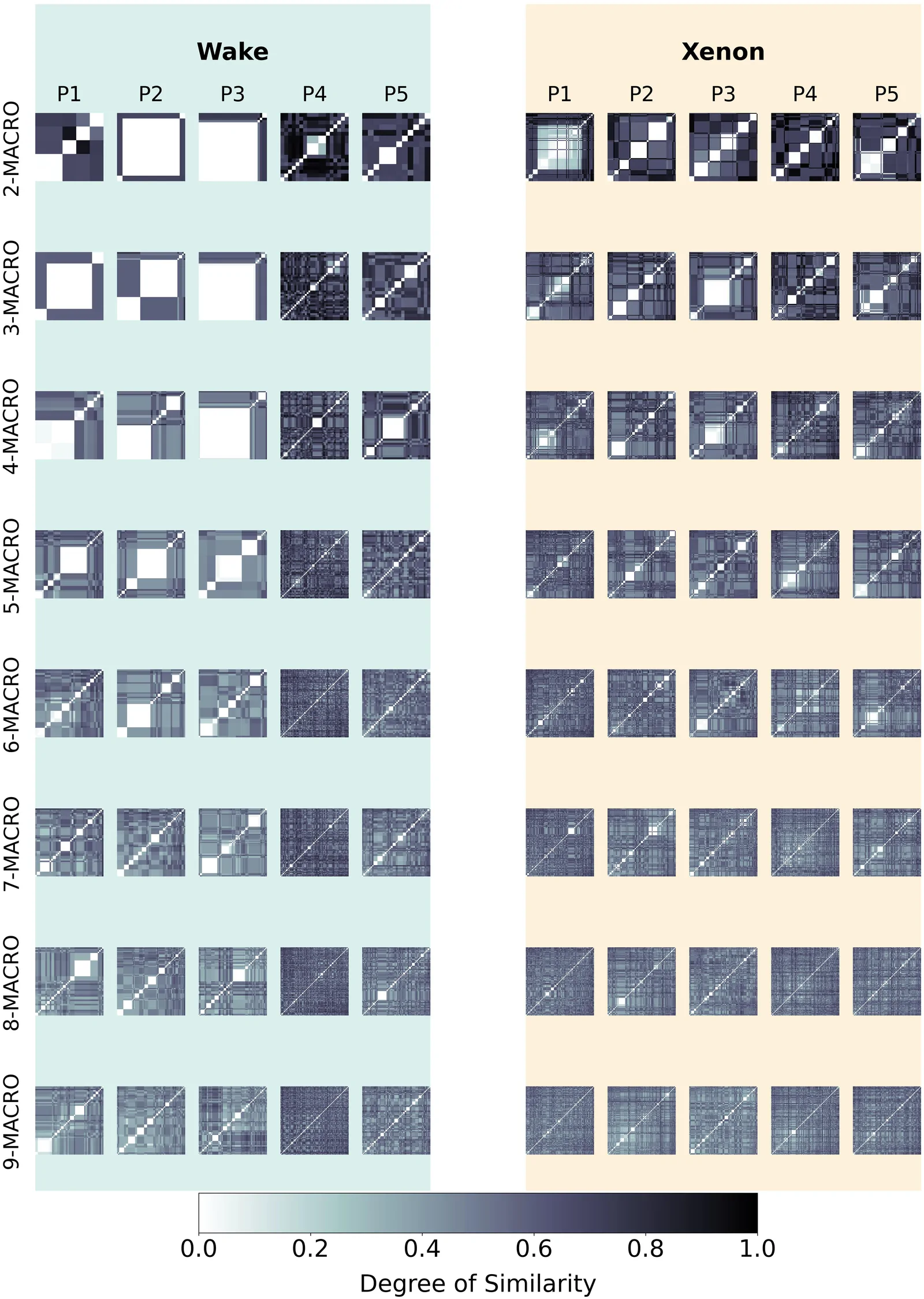
Xenon-induced changes in emergent dynamical structure on higher-order spatial scales. As in Figure 2, similarity matrices display pairwise subspace–angle distances between *n*-macros across optimisation runs during Wake (left) and Xenon (right) conditions.

Xenon, another anaesthetic condition associated with unresponsiveness and absence of reportable experience, compared with wakefulness, produced significant fragmentation in emergent structure across all scales (Fig. 5), with increasing divergence at higher scales.

**Fig. 6:**
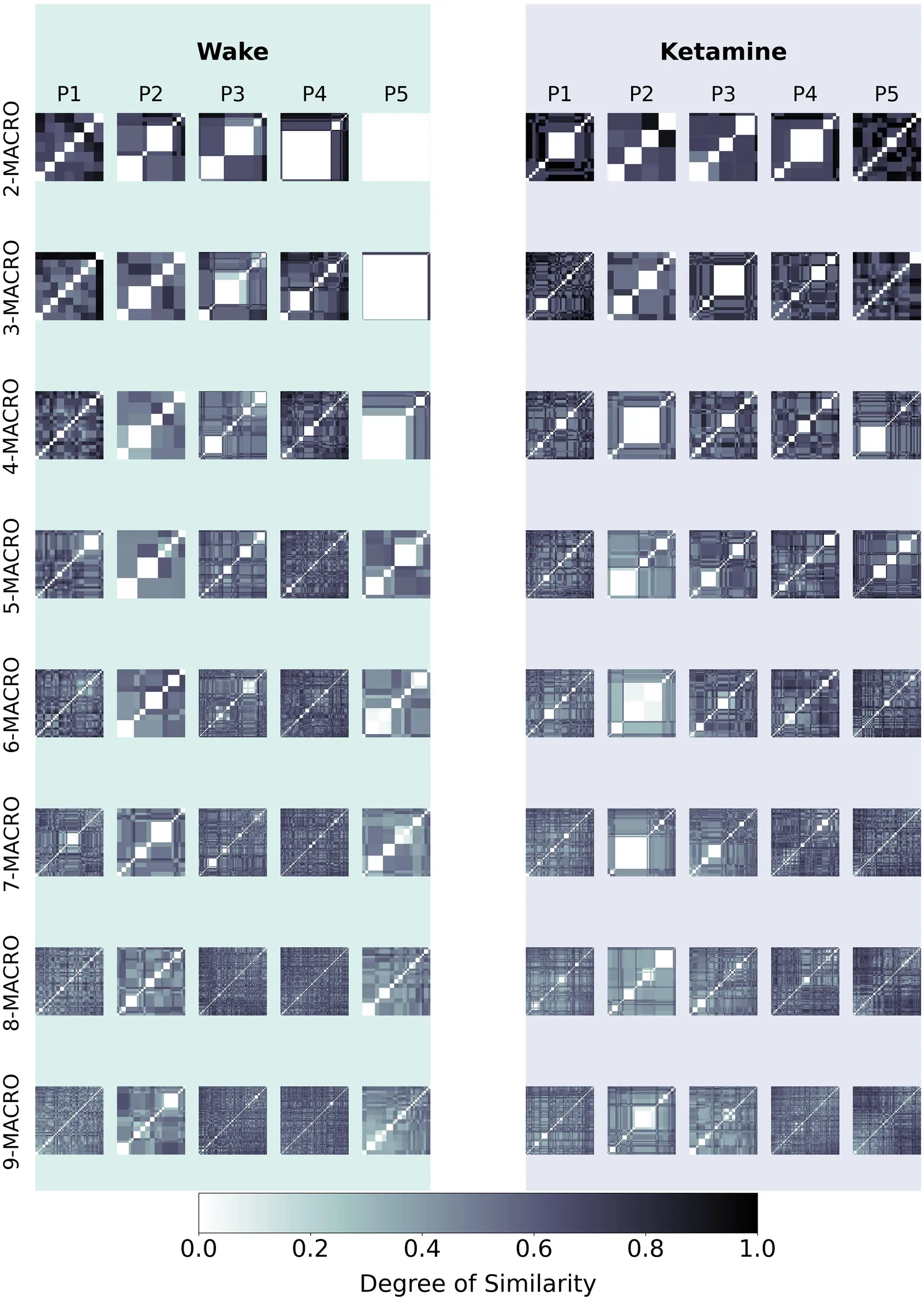
Ketamine-induced changes in emergent dynamical structure on higher-order spatial scales. As in Figure 2, similarity matrices display pairwise subspace–angle distances between *n*-macros across optimisation runs during Wake (left) and Ketamine (right) conditions.

Finally, under ketamine (Fig. 6), the emergent structure more closely resembled that of the wakefulness. Blocks of convergence in optimisation runs persisted across most higher-order scales, indicating relatively stable and reproducible macroscopic organisation. Notably, ketamine is associated with altered but reportable subjective experience rather than complete suppression, and the relative preservation of macroscopic structure aligns with this phenomenological distinction (Sarasso et al., 2015).

To quantify these patterns, we introduce a *Structure*–*Fragmentation (SF) index* defined as the proportion of near-zero similarity values (<0.0001 ) within each 100 × 100 similarity matrix across the eight macrolevels. Higher SF values, therefore, indicate greater recurrence of similar macroscopic solutions and stronger dominance within the optimisation landscape.

Using repeated-measures ANOVA with *subject* as a random factor, we tested for main effects of macroscale, condition (Wake vs Anaesthesia), and their interaction, followed by FDR-corrected post-hoc Wilcoxon tests to determine the directionality of the drug-induced effects.

**Fig. 7:**
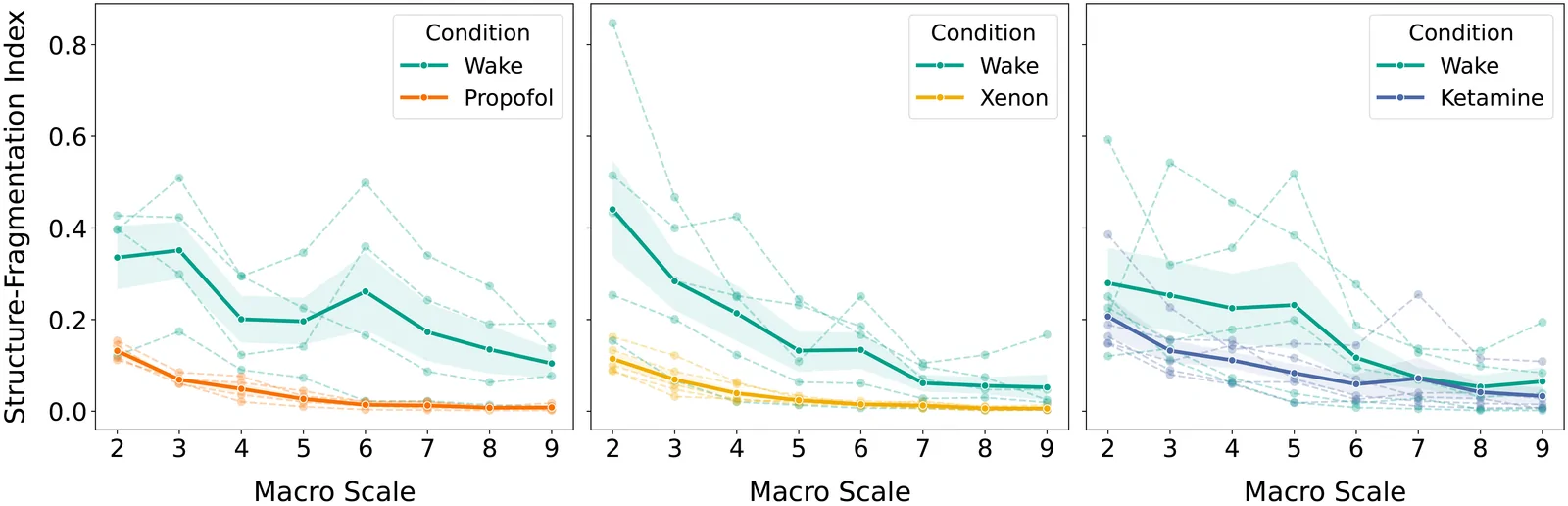
Emergent dynamical structure across wake and anaesthetic conditions (propofol, xenon, ketamine). Each panel displays the proportion of near-zero similarity values (<0.0001 ) across 100 optimisations at each macroscale (2≤n≤9 ) for individual subjects under wakefulness (teal) and one anaesthetic condition. We consider this the *Structure*–*Fragmentation (SF) Index* of the emergent dynamical landscape. Dashed lines connect points from the same subject, allowing within-subject comparisons across conditions and scales. Group-level means are overlaid with 90% confidence intervals. Across all panels, wakefulness consistently exhibits a higher SF index (proportions of near-zero similarity values), indicating greater structure in emergent macroscopic landscape. Under propofol (left), a marked reduction in SF index is observed, particularly at intermediate-to-coarse scales, suggesting widespread fragmentation and instability in the emergent dynamical landscape. Xenon (middle) similarly shows degraded structure relative to wake, with pronounced divergence at higher-order scales (*n* ≥ 7), reflecting scale-selective disruption and fragmentation of emergent organisation. In contrast, under ketamine (right), the structure of the emergence landscape is relatively preserved, with trajectories closely tracking the wake condition across all scales. Although no macroscales survived FDR correction in post-hoc comparisons, the consistent directional trend of wake > anaesthesia is compatible with the hypothesis that emergent structure is more stable and organised in conditions permitting conscious experience.

Across all three anaesthetic agents—propofol, xenon, and ketamine—we observed highly significant main effects of macroscale on the SF index (Fig. 7). Emergent structure varied systematically across spatial scales in all cases: propofol (F(7,21)=9.99 , p<.0001 , $\eta^{2}=0.769$ ), xenon (F(7,28)=16.38 , p<.0001 , $\eta^{2}=0.804$ ), and ketamine (F(7,28)=15.35 , p<.0001 , $\eta^{2}=0.793$ ). These findings confirm that emergent structure is intrinsically scale dependent, independent of pharmocological condition (see Fig. 7).

The main effect of condition (wakefulness vs anaesthesia) was significant for propofol (F(1,3)=10.65 , p=.0470 , $\eta^{2}=0.780$ ), significant for xenon (F(1,4)=9.16 , p=.0389 , $\eta^{2}=0.696$ ), and not significant for ketamine (F(1,4)=2.52 , p=.188 , $\eta^{2}=0.386$ ). These results indicate that propofol and xenon significantly reduce macrolevel dominance within the emergent dynamical landscape relative to wakefulness, whereas ketamine does not produce a reliable global difference.

Most notably, the macro × condition interaction was significant for xenon (F(7,28)=5.65 , p=.0004 , $\eta^{2}=0.585$ ), showed a trend for propofol (F(7,21)=2.42 , p=.056 , $\eta^{2}=0.446$ ), and was non-significant for ketamine (F(7,28)=1.21 , p=.332 , $\eta^{2}=0.232$ ). These results indicate that xenon, and to a lesser extent propofol, modulates emergent structure in a scale-selective manner.

In these regimes, the SF index is substantially higher during wakefulness than anaesthesia, particularly under xenon and propofol. This suggests that wakefulness is characterised by more reproducible and structurally dominant macroscopic process, especially at coarse levels of description (see Supplementary Fig. S4 and Supplementary Text S4 for a less conservative Kolmogorov–Smirnov (KS) test).

We performed the same scale-resolved interaction analyses for the SF index as we did for the DD results above. First, a three-agent mixed ANOVA was run at each macroscale with condition (Wake vs Anaesthesia) as the within-subject factor and anaesthetic agent as the between-subject factor. This model tests whether the anaesthesia-induced SF change differs anywhere among the three agents. The three-agent condition × agent interaction did not survive FDR correction at any macroscale (F(2,11)=0.18 –3.13, p=.084 –.834, q=.412 –.834, $\eta_{p}^{2}=.032$ –.363; Supplementary Table S3).

We then ran planned pairwise 2 × 2 mixed ANOVAs for each drug pair and macroscale. No pairwise condition × agent interaction survived FDR correction. Propofol and xenon did not differ in their SF condition effects at any scale (F(1,7)=0.08 –2.84, p=.136 –.783, q=.581 –.783, $\eta_{p}^{2}=.012$ –.288; Supplementary Table S4). Propofol and ketamine also did not differ significantly at any scale (F(1,7)=0.03 –4.12, p=.082 –.866, q=.216 –.866, $\eta_{p}^{2}=.004$ –.371; Supplementary Table S4), nor did xenon and ketamine (F(1,8)=0.08 –2.72, p=.138 –.780, q=.534 –.780, $\eta_{p}^{2}=.010$ –.254; Supplementary Table S4). Thus, although within-agent SF reductions were observed for propofol and xenon, direct interaction tests did not show statistically reliable differences in the magnitude of the SF condition effect between anaesthetic agents.

Together, these findings demonstrate that while dynamical independence (low DD) may increase under anaesthetic agents, the structural stability of the emergent macroscopic landscape can simultaneously degrade, or fragment. Relative to wakefulness, propofol and xenon are each associated with reductions in the SF index, suggesting fragmentation of the emergent landscape. Relative to wakefulness, ketamine does not show a significant global reduction in SF index, consistent with relative preservation of emergent structure. Direct scale-resolved condition × agent interaction tests further showed that the magnitude of SF change was not statistically distinguishable between anaesthetic agents, indicating that apparent differences between drug-specific SF profiles should be interpreted descriptively rather than as established between-drug effects. Thus, the principal inference from the SF analysis is that wakefulness supports a more reproducible and macroscopically organised emergent landscape, whereas anaesthesia is associated with fragmentation of this landscape.

This dissociation between degree of dynamical dependence and the structure of the emergent landscape underscores that emergence, in our quantitative sense, does not necessarily imply globally dominant multiscale organisation.

### 3.3 Source contributions to emergent *n*-macros

**Fig. 8:**
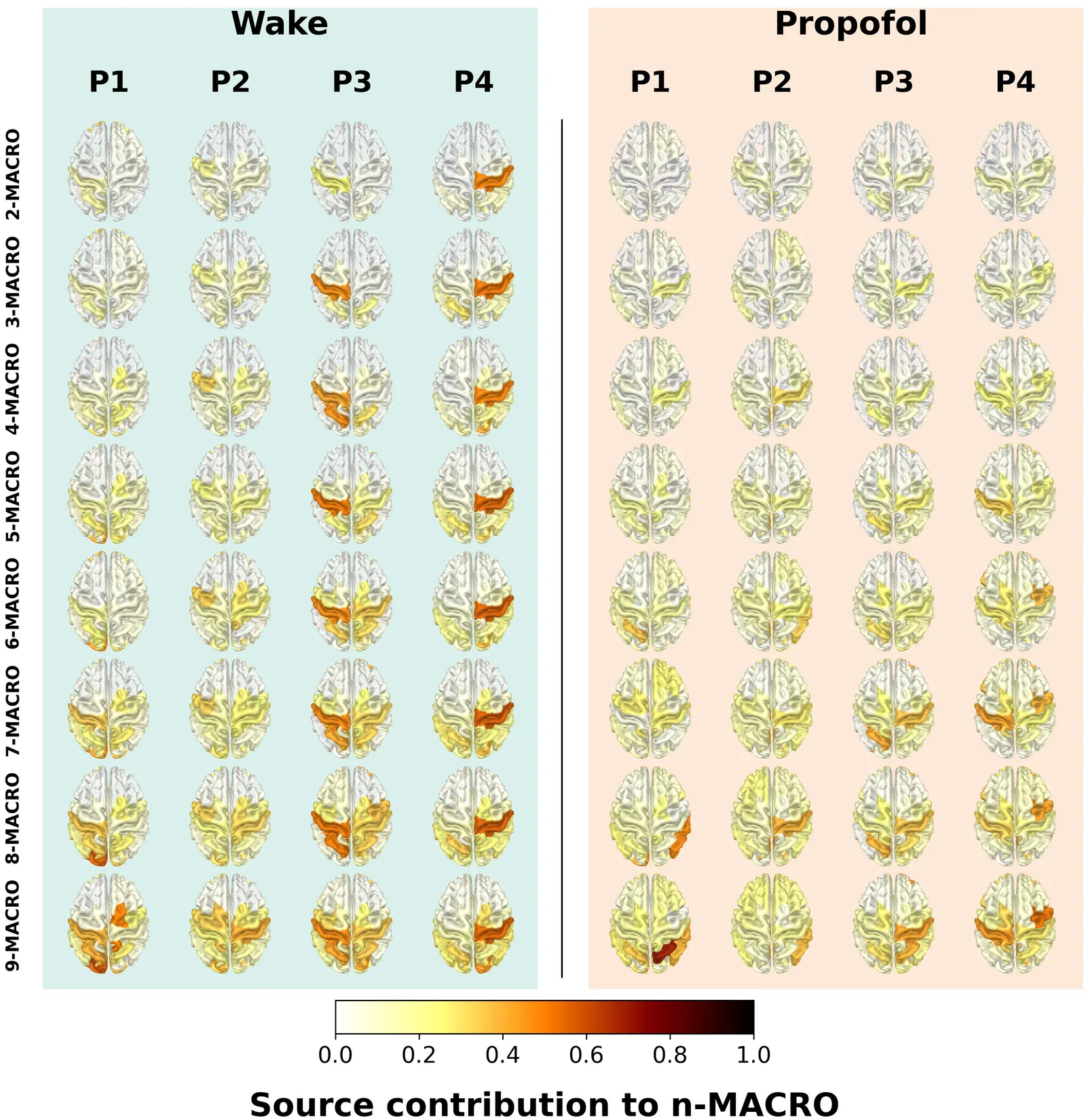
Source contribution to higher-order interactions in propofol v wake. Single-region contributions per macro across subjects during propofol-induced anaesthesia and corresponding wake states. Each row represents an *n*-macro, and each column represents an individual subject. Colour intensity reflects the beta statistic (within the range [0, 1]), indicating the strength of each source’s contribution to the *n*-macro above chance. Zero equates to contributions expected by chance. The higher the values the stronger the contribution (refer to Methods Section 2.5.2 for details on beta statistic computation.

**Fig. 9:**
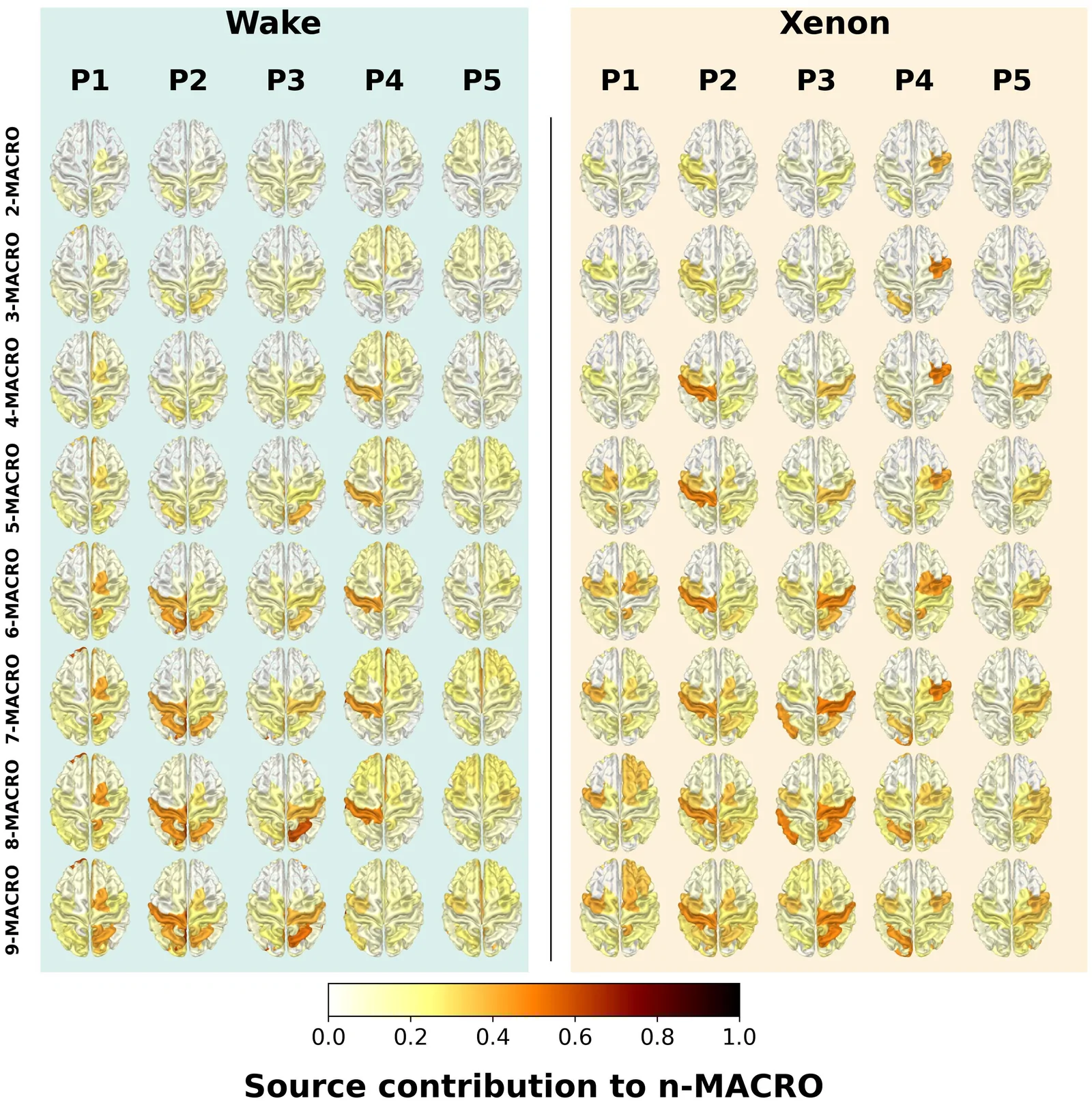
Source contributions in xenon v wake. As in Figure 6, but for xenon-induced anaesthesia and wake.

**Fig. 10:**
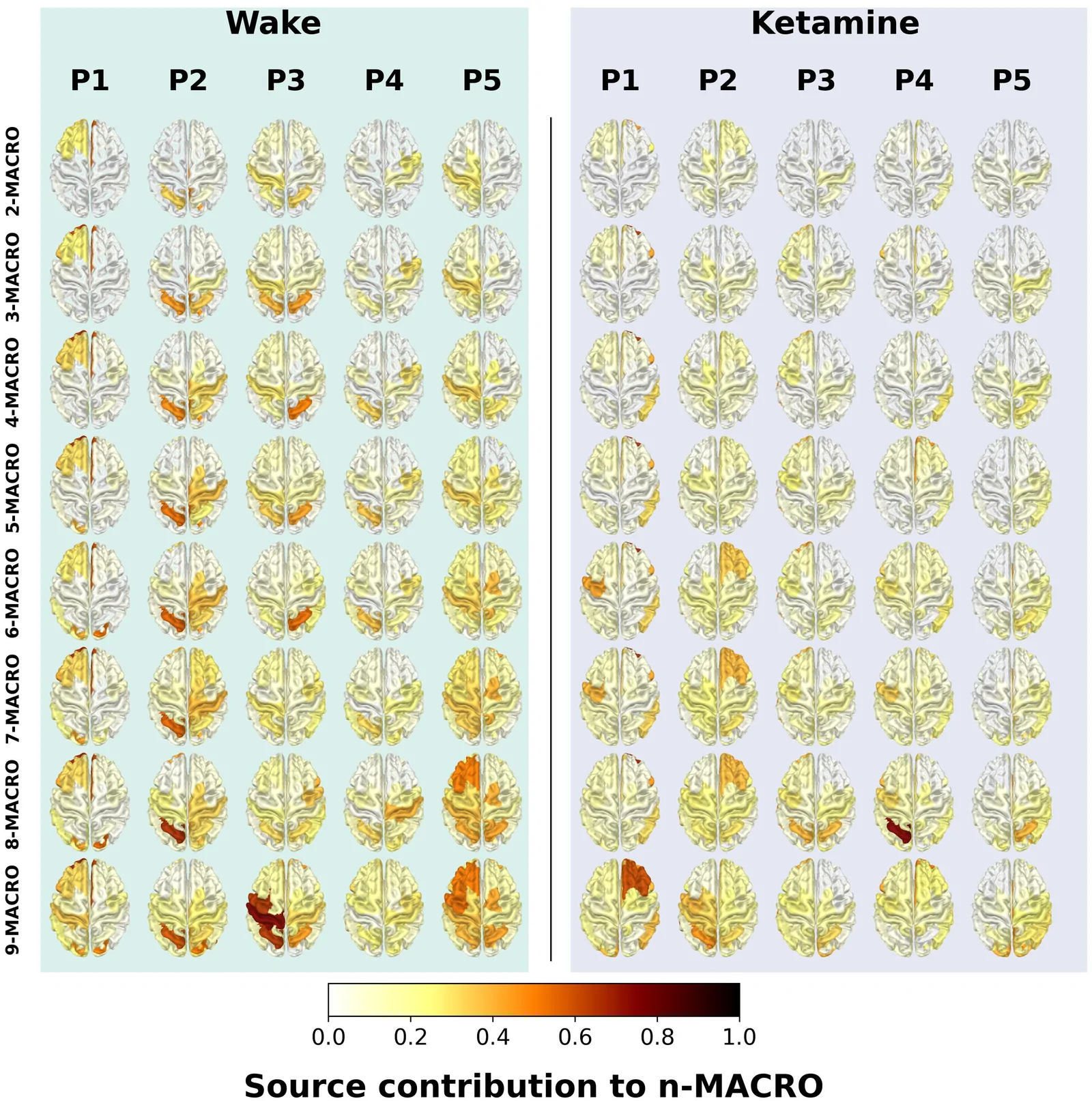
Source contribution in ketamine v wake. As in Figure 6, but for ketamine-induced anaesthesia and wake.

As an exploratory analysis, we next computed the contributions of individual sources to the *n*-macros to better understand the relationship between the higher-order interactions captured by *n*-macros and the lower-scale sources from which they emerge. Figures 8, 9, and 10 illustrate the anatomical source contributions to emergent *n*-macros above what would be expected by chance (refer to Methods Section 2.5.2 for details on how we computed the source contributions and Methods Section 2.8 for statistical analysis).

Wakeful participants often exhibit well-defined sets of sources of high contribution that persist or intensify at higher *n*-macroscales, reflecting a spatially localised contribution of specific sources to emergent *n*-macros (Fig. 8). Notably, participants P2, P3, and P4 display distinct localised patterns of strong contributions that overlap with the parietal cortex and extend across multiple scales. In two participants (P1 and P2), this contribution from the posterior and parietal regions appears more pronounced in higher scales. In contrast, the same participants under propofol show that these spatially concentrated patterns are less evident, yet a similar set of sources with high contribution over posterior regions is observed across higher spatial scales (>6), which closely resembles wakefulness but is diminished in intensity. In general, the contributions appear more uniform with no clear prominence of specific sources. We note that in wakefulness, P3 and P4 exhibit strong lateralisation across the motor cortex, and the pattern becomes less pronounced and more bilateral with increased higher scales.

Compared with wakefulness, xenon does not show a systematic increase in source-level β-contributions across higher-order scales. This differs from the pattern observed under propofol, where source contributions to higher-order scales showed relatively greater β-contributions than wakefulness. We note that this description refers to within-condition contrasts relative to wake and should not be interpreted as a formal statistical comparison between anaesthetic agents. In both conditions, source contribution patterns vary across participants, and considerable inter-subject variability is evident. Overall contributions tend to increase with scale and are similarly concentrated in the parietal cortex for both, wakefulness and xenon. However, in two out of five participants, frontal source contributions are reduced under xenon relative to wake. Some lateralisation is also evident across individuals in both states, though without a consistent hemispheric bias across the sample. These results suggest that while xenon preserves aspects of the posterior dynamics observed during wakefulness, this activity may be weaker, with reduced frontal contributions helping to distinguish the two states. Given the spatial resolution of source-reconstructed EEG, these observations should be interpreted descriptively rather than as definitive inferences about source-level contributions. The broader implications of these findings are discussed in the Discussion section.

Lastly, compared with wakefulness, ketamine shows a different pattern of source contributions across higher-order scales. While wake continues to exhibit increasing source contributions with scale, typically concentrated over posterior regions (with the exception of participant P1), this pattern is less pronounced under ketamine. Specifically, ketamine displays lower overall increases in source contributions across scales and greater spatial heterogeneity. Notably, three out of five participants (P1, P2, and P4) show increased contributions in frontal regions under ketamine, contrasting with the posterior focus seen in wake. However, this pattern is not uniform across participants. These findings suggest that ketamine differs from wakefulness not only in the degree of emergence and emergent dynamical structure, but also in the spatial localisation of their underlying source contributions.

Before proceeding to group-level comparisons across conditions, we note that the individual-level statistical analysis revealed that several regions showed significant contributions to *n*-macros after correction for multiple comparisons. These significant patterns are visualised in Supplementary Figures S5–S7, which provide participant-level cortical maps for each condition, and each macroscale. Thus, although the following group-wise comparisons face stricter correction burdens, clear significant and localised source-level effects are already evident at the single-scale, single-subject level.

To complement the single-subject analysis, we evaluated group-level differences using a similar statistical approach as with the DD values in Figure 2. Specifically, we applied the grouped Mann–Whitney U-test separately to each source *i*, allowing us to determine whether the source’s contribution (β-scores) to an *n*-macro differs significantly between wakefulness and anaesthesia. As before, the test first compares each participant’s wakeful data against the corresponding anaesthetic data and then aggregates (across participants in the same anaesthetic condition) the U statistics and Z-scores using the methods defined in subsection, *Statistical Comparison of β-value Values*, of the Methods Section 2.8. Notice that now the tests are *not* independent across sources, and thus we employed an FDR-based multiple comparison correction that assumes dependence of hypotheses.

**Fig. 11:**
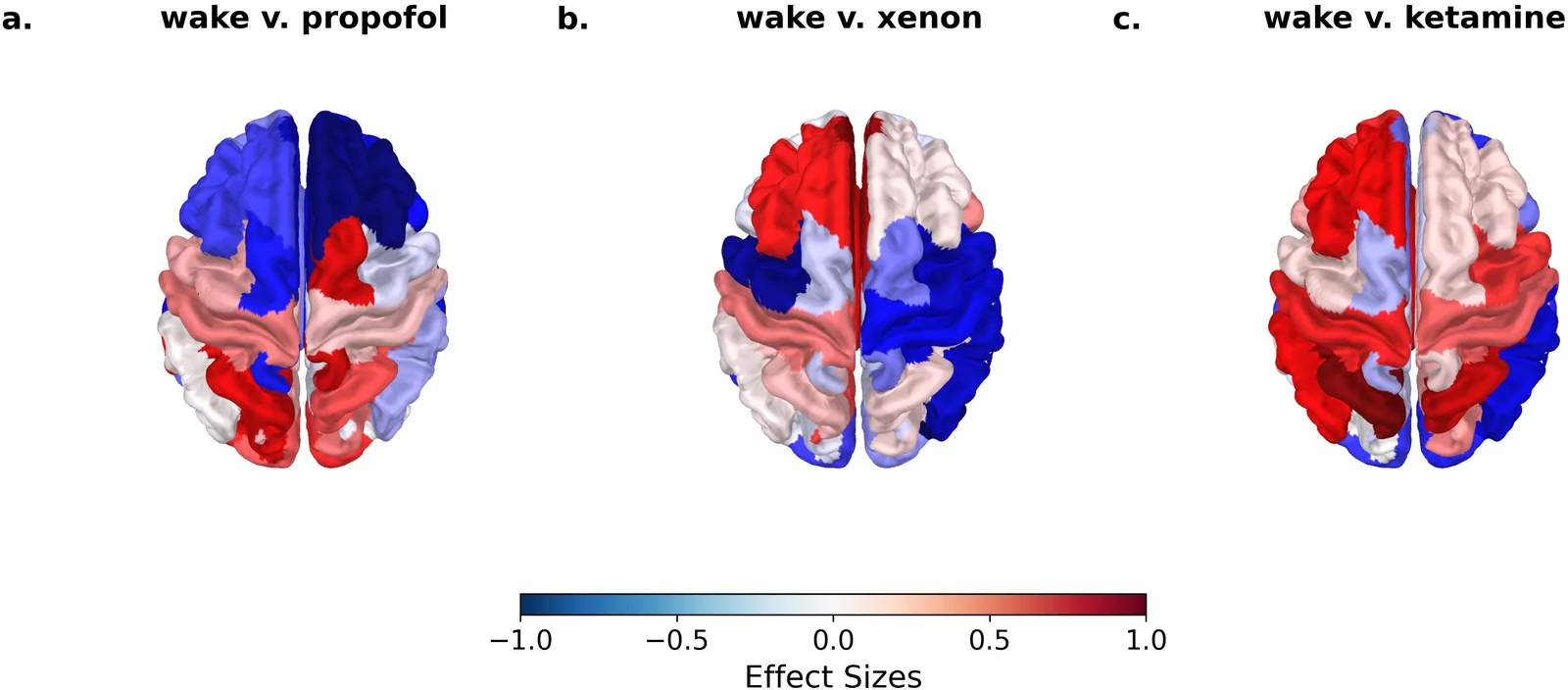
Contrasts between source contributions across all *n*-macros and conditions. Source contributions to *n*-macros across higher-order scales (2≤n≤9 ) in wakefulness compared with (a) propofol, (b) xenon, and (c) ketamine. Red denotes regions where wake exhibits greater β-values than the anaesthetic condition, while blue indicates greater contributions under anaesthesia. Region-wise wake versus anaesthetic contrasts were assessed using a grouped (participant-stratified) Mann–Whitney U test, aggregating subject-specific comparisons and converting to asymptotic Z-scores. Dependency-robust FDR correction was applied across the 46 regions. After correction, no region survived group-level significance. The test evaluates stochastic dominance of regional β-distributions in wake relative to the corresponding anaesthetic condition.

Figure 11 visualises the resulting effect sizes: red indicates sources contributing more strongly to *n*-macros during anaesthesia, while blue highlights sources with stronger contributions under wakefulness. Although these effect size differences suggest potential distinctions in how each anaesthetic condition alters the spatial profile of emergent dynamics, none of these effects survived correction for multiple comparisons and should, therefore, be interpreted with caution. Nevertheless, the significant individual-level patterns (Supplementary Figs. S5–S7 and Supplementary Texts S5–S8) provide complementary evidence of source-specific reorganisation across states.

Compared with wakefulness, all anaesthetic conditions show alterations in the spatial distribution of source contributions to higher-order dynamics. For propofol, participants during wakefulness show that contributions are concentrated around posterior regions (Fig. 11a), consistent with individual-level patterns. Under propofol, these contributions appear to shift towards frontal regions, as suggested by negative effect size values. In the xenon condition, posterior contributions during wake are not as prominent, and xenon introduces greater lateralisation and additional contributions from frontal and parietal sources (Fig. 11b). For ketamine, source contributions appear focused in posterior areas during wake, but with reduced frontal involvement under ketamine relative to wake (Fig. 11c). However, substantial between-subject variability, particularly evident even during wakefulness, highlights the need for larger sample sizes to more robustly characterise these patterns.

## 4 Discussion

The present study quantified emergent multiscale dynamical organisation across multiple spatial scales in source-reconstructed EEG under three pharmacologically distinct anaesthetic interventions. The degree of emergence was computed using dynamical independence and we introduced here the SF index to characterise the fragmentation of the corresponding emergent landscape. The SF index specifically captures the stability and reproducibility of emergent n-macros across macroscopic spatial scales. In an exploratory analysis, we also assessed the spatial embeddedness of these n-macros to further characterise the structure of emergent dynamics across wakefulness and three anaesthetic agents. As detailed in the Methods section, all main statistical contrasts are between each anaesthetic condition and its matched wakefulness baseline. A supplementary analysis was used to assess between-subject differences across anaesthetic agents.

Within this scope, our findings indicate that across macroscopic scales and compared with wakefulness, propofol and xenon were characterised by higher emergence (lower dynamical dependence), while ketamine (higher dynamical dependence) was characterised by reduced emergence. Together, these drug-induced changes in patterns suggest that wakeful conscious processing depends not only on integration within a single scale, but also on integration across scales, a finding we develop below.

Given the common conflation of complexity and emergence in the neuroscience literature, it is understandable that one might intuitively expect indices of emergence to track with signal diversity (M. Schartner et al., 2015) and algorithmic complexity (Cofré & Destexhe, 2025) which are often heightened during wakefulness and reduced under anaesthesia (Casali et al., 2013; M. Schartner et al., 2015; M. M. Schartner et al., 2017). However, we caution against this assumption; algorithmic complexity and emergence represent fundamentally different aspects of system organisation.

Within the DI framework, emergence is operationalised through dynamical closure rather than complexity. This distinction is important for interpreting our results. A distinctive feature of DI is its explicit characterisation of dynamical scales and their corresponding (in)separability from the microlevel base. Unlike global complexity measures (e.g., Lempel–Ziv complexity), modularity metrics, or scalar information-theoretic summaries, which are generally agnostic about dynamical scale, DI evaluates dynamical closure as a function of scale—in terms of dimensions of the macroscopic variable. This enables identification of specific macroscopic processes on candidate spatial scales at which they become more or less separable from their microscopic bases. Because brain dynamics unfold across multiple interacting scales rather than at a single level of description (Goldman et al., 2019, 2023; Sacha et al., 2025), such scale-resolved characterisation provides insight into multiscale organisation that cannot be obtained from scale-agnostic measures.

In the dynamical-closure framework, a process is deemed emergent once its future can no longer be improved by considering the past of its microscopic constituents. Crucially, this criterion speaks only of independence from the lower-level base, not of how well the macro predicts itself. An *n*-macro can, therefore, be perfectly emergent yet *internally* entropic, indeed, it might look like near-white noise. What matters for functional relevance, however, is whether such macros also exhibit coherent *internal* dynamics. Although the present study focuses on an aspect of emergent dynamical structure by way of the emergent landscape revealed by stable and dominant *n*-macros (Figs. 4–6), we did not evaluate the intrinsic structure of each macro, leaving that question for future work.

This shift of focus from the degree of emergence to aspects of emergent dynamical structure motivates our two-part analytic strategy: we quantify *(i)* the degree of emergence (closure from the micro-level) and *(ii)* the organisation and structure of the emergent landscape across candidate spatial scales. Treating both facets as complementary is essential for a full account of emergence in brain dynamics.

In light of this, it is most informative to view our findings through the lens of emergent *structure*. Simply, differences in dynamical (in)dependence must be interpreted alongside the structure of the emergent landscape, rather than in isolation. While propofol and xenon exhibit higher emergence, they lack dominant *n*-macros across scales, implying fragmented dynamical organisation. Conversely, wakefulness (and, to a lesser degree, ketamine in comparison with wake) shows dominant, recurring *n*-macros that point to a structured, and more organised, emergent landscape. We, therefore, developed the SF index—quantifying the frequency with which an *n*-macro re-emerges across optimisation runs—as a practical proxy for robust higher-order organisation.

Within the optimisation landscape, multiple *n*-macros can co-exist at any scale; fragmentation signals not competition among alternatives but the absence of a deep basin of attraction. Repeated recovery of the same *n*-macro indicates such a basin and thus dominant dynamics, whereas scattered minima point to a disorganised, noisy landscape. We visualise these basins with similarity-matrix “white” blocks, enabling our extended framework to separate structured emergent landscapes from high-dimensional noise, using a simplified SF index, and to pinpoint candidate neural correlates of conscious state.

A common view is that consciousness “emerges” from brain activity, which might suggest that conscious wakeful states should score higher on measures of emergence anaesthetic-induced ones. Our findings challenge any such straightforward identification of “more emergence” with “higher” conscious level. In the DI framework, emergence is defined formally by the degree to which dynamical subsystems are self-contained across scales: a property of scale-(in)separability, not a normative index of cognitive, behavioural, or experiential richness, per se. Propofol and xenon each exhibit high DI but fragmented emergent landscapes (low SF) relative to wakefulness, whereas wakefulness shows lower independence yet a more structured emergent dynamical landscape. For ketamine, the main effect of condition relative to wakefulness did not reach significance for the SF index relative; we, therefore, interpret its emergent landscape as not reliably distinguishable from the wake condition. These findings suggest that the degree of emergence is only part of the story; macroscopic processes may need to remain partially coupled to their microscopic dynamical base to sustain stable and dynamically relevant large-scale organisation.

A crucial implication follows. Conscious states appear to eschew a clean *separation of scales* between micro and macro—the kind that would be reflected in near-perfect dynamical closure (near-zero DD). Rather than full dynamical independence, wakefulness operates in a *scale-integrated* manner, where multiple levels of description remain mutually entangled with the microscopic base. This contrasts sharply with engineered systems such as computers, where a strict hardware–software separation of scales is enforced by design precisely because it makes construction tractable. Evolved biological systems, it seems, work differently; in certain states, such as wakeful consciousness, they sacrifice separability for integration across scales (Aru et al., 2020, 2023; Goldman et al., 2019; Milinkovic & Aru, 2025; Pessoa, 2023; Seth, 2025). Where and how this principle of scale integration might bear on minimal consciousness in synthetic systems remains an open and, we think, exciting question.

The ketamine condition may further speak of the relationship between multiscale emergent organisation and consciousness. Despite showing significantly higher DD than wakefulness, indicating increased scale integration, ketamine did not show a significant global reduction in SF, suggesting relative preservation of emergent macroscopic structure *and* is accompanied by vivid, dream-like reports (Sarasso et al., 2015). This alignment between emergent structure and subjective experience under ketamine further supports the proposal that dynamical structure and scale integration, rather than level of emergence alone, may be better predictive markers of consciousness. Consistent with this interpretation, direct scale-resolved interaction tests showed that ketamine differed significantly from xenon in its DD condition effect across all macroscales, whereas no significant between-agent interaction was observed for SF. This suggests that ketamine and xenon may differ specifically in how they alter micro-to-macro dynamical dependence, even if they share broader anaesthetic effects on neural dynamics. In this respect, our findings extend recent work showing convergent destabilisation of neural dynamics across anaesthetic agents (Eisen et al., 2026), by indicating that emergence-based measures can reveal agent-specific differences in cross-scale organisation. These results, therefore, suggest a “sweet spot” of emergence, whereby macroscopic processes are sufficiently self-contained in order to form a structured multiscale dynamical landscape, yet, are still relatively integrated with their micro-level sources. Direct testing of this hypothesis requires between-drug designs with adequate statistical power. Future work should extend these findings by jointly evaluating each macro’s degree of dynamical closure—as we do here—*and* its self-predictive power, in order to determine its functional relevance. Many other interesting questions arise from this: for example, does scale integration also peak at this putative “sweet spot”, and how do these dynamical signatures relate to other powerful features of scale relations in complex systems, such as criticality and critical regimes (Barnett et al., 2013; Di Volo & Destexhe, 2021).

Although group-level source contribution effects did not survive correction for multiple comparisons, individual maps (Supplementary Figs. S5–S7) revealed suggestive, drug-induced spatial patterns. In particular, during wakefulness, dominant *n*-macros frequently involved posterior regions, including occipital cortex, precuneus, and posterior cingulate. These areas have previously been associated with conscious content in sleep and dreaming studies (Pigorini et al., 2015). Alterations in slow-wave activity within this posterior network, which reflect changes in neuronal synchrony and cortico-cortical connectivity (Achermann & BorbÉly, 1998; Massimini et al., 2005; Siclari et al., 2017), have been linked to the presence or absence of subjective experience during sleep.

We emphasise that, given the spatial limitations of source-reconstructed EEG and the absence of group-level corrected effects, these patterns should be interpreted as exploratory and hypothesis generating rather than as definitive localisation findings. Nonetheless, the observed posterior predominance during wakefulness and its relative attenuation under propofol (alongside increased frontal contributions consistent with the frontal alpha shift (Koskinen et al., 2001; Purdon et al., 2013)) suggest that the anatomical distribution of emergent dynamical structure may vary systematically across brain states.

Taken together, these preliminary data suggest that the anatomical localisation of structured emergent dynamics shifts with state. Specifically, posterior cortices may dominate in conscious conditions, whereas deep anaesthesia may recruit more anterior generators. Because the sample size was modest and inter-individual variability substantial, higher-resolution techniques (MEG, sEEG, iEEG) will be required to confirm whether posterior hot-zone engagement is a reliable signature of scale-integrated macros in conscious brains.

But, we hold that the non-significant results at the source level and the diffuse nature of the contributions to *n*-macros are indicative of an even deeper trend. That is, projecting the macroscopic processes back to the source level to capture the contribution from each individual region does not provide us with a localised cluster over which the macrovariable resides. If this were the case, then, merely applying cluster analyses of networks would suffice. It appears that higher-order dynamics resist this reduction to their microlevel constituents, evincing support for studying emergence, empirically, in neural systems. This will help us understand completely novel features neural dynamics, such as their multiscale organisation and integration.

### 4.1 Comparison with alternative approaches to higher-order interactions

By going beyond solely measuring the quantity of emergence, our method addresses the broader challenge of defining the multiscale functional organisation of biological brains directly from data. Building on earlier ideas (McCulloch, 1945) and recent developments in neuroscience (Semedo et al., 2019) and biology (McMillen & Levin, 2024), we emphasise that global brain states (particularly of consciousness) involve reorganisation of the emergent multiscale structure of brain dynamics.

Neural activity has previously been shown to organise into low-dimensional subspaces (Ebitz & Hayden, 2021; Langdon et al., 2023; Perich et al., 2025; Thibeault et al., 2024). Our study builds on this work by providing a principled way to identify emergent dimensionally reduced macroscopic processes directly from neurophysiological data and measure their degree of emergence in information-theoretic quantities. To that end, our findings suggest a heterarchical view of brain organisation, consistent with the idea that higher-order scales remain partially dependent on, and distributed across lower-level dynamics (McCulloch, 1945).

Our approach naturally invites comparison with other information-theoretic measures of emergence designed for neuroscientific data (Hoel et al., 2013; Rosas et al., 2020), some of which we have already touched upon above. Specifically—for instance—synergy-based causal emergence is an alternative perspective on emergence which focuses on the extra information provided by jointly distributed variables in predicting future states beyond what is carried uniquely by the individual variables themselves (Mediano et al., 2021; Rosas et al., 2020; N. Timme et al., 2014; N. M. Timme & Lapish, 2018; Williams & Beer, 2010). These methods have computed the synergistic capacity present in neural data in various contexts, including neurodegeneration (Herzog et al., 2022), ageing (Gatica et al., 2021), resting-state fMRI in humans and macaques (Luppi et al., 2022; Varley, Pope, Faskowitz, et al., 2023; Varley, Pope, Grazia, et al., 2023), neural spike dynamics (Stramaglia et al., 2021), and mouse neocortical cultures (Sherrill et al., 2020). However, despite recent algorithmic advances, the measures based on a partial information decomposition mentioned in the introduction still face practical constraints when they are extended beyond higher-order sets larger than *n* = 5 (Jansma et al., 2024), necessitating secondary graph-theoretic strategies to glean insights from higher-order interactions such as those used in Luppi et al. (2022) and Varley, Pope, Faskowitz, et al. (2023). In this sense, synergy can be seen as a specific aspect of emergence (Luppi et al., 2021, 2023, 2024; Mediano et al., 2022), distinct from the dynamical closure at the heart of our DI-based definition of emergence.

Further, unlike alternative motivations for coarse graining, such as clique and motif network structure, or simplicial complex identification, which are graph-based definitions of higher-order interactions (Battiston & Petri, 2022; Rubinov & Sporns, 2010), *n*-macros serve as lower-dimensional subspaces that capture relevant multivariate dynamics. Conceptually similar to principal components (see Methods Section 2.5) and neural manifolds, *n*-macros highlight how multiple variables (for instance, brain regions) collectively interact, potentially offering a fresh perspective on *communication subspaces* (Humphries, 2020; Semedo et al., 2019). The definition or role of *n*-macros as communication subspaces is beyond the scope of the current paper, however, this is an active line of research we are currently pursuing.

A key aspect for how our DI-based approach can complement the above-mentioned synergy- and network-based perspectives is by identifying emergent macroscopic processes directly from data rather than relying on predefined observables. Furthermore, because DI scales reasonably well with both system dimension and by virtue of the reduced dimensionality of *n*-macros, this approach circumvents some of the computational constraints inherent in partial information decomposition methods (Gutknecht et al., 2021; Makkeh et al., 2025). Although recent work using *O-information* (Herzog et al., 2024; Rosas et al., 2019; Varley, Pope, Faskowitz, et al., 2023) seeks to overcome these constraints in larger systems, the direct discovery of *n*-macros across scales via DI offers a distinct advantage by capturing the multiscale integration and organisation in high-dimensional neural data.

### 4.2 Limitations and future directions

This study quantifies the structure of emergent dynamics by tracking *(i)* how strongly *n*-macros dominate higher-order scales (via similarity matrices) and *(ii)* where those macros localise over brain sources. Although EEG is sufficient to reveal scale-dependent emergent structure, its spatial precision is inherently limited due to volume-conduction artefacts, even after source reconstruction. MEG, ECoG, or sEEG/iEEG will be better suited for fine-grained spatial localisation. In light of this, we address these methodological considerations explicitly below to facilitate reproducibility and guide future applications of our pipeline.

#### 4.2.1 Robustness of DI to source reconstruction properties

Because DI is computed from fitted multivariate autoregressive (VAR) models of the reconstructed regional time series, its behaviour depends on the estimated covariance structure and predictive relationships between sources. Signal-to-noise ratio (SNR) limitations in the raw recordings may inflate residual variance, potentially increasing estimated DD values (and hence DI). However, such an effect would bias conservatively against detecting high DI and has no effect on falsely inferring the existence of “artificial” macroscopic variables. It should be noted that this limitation stands for comparisons across recordings, but for single-session recordings, any recording-driven SNR inflates both full and reduced model residual variances, so it is not given that their log-ratio (the GC) will increase.

Spatial leakage from the inverse solution can introduce artefactual correlations between neighbouring regions, particularly when analyses are performed at fine-grained voxel resolution or using high-dimensional parcellations. In the context of DI, such leakage would tend to increase apparent micro–macro coupling, thereby conservatively elevating DD values. Consequently, leakage would bias results towards reduced apparent emergence (i.e., higher DD), rather than artificially generating spurious macroscopic independence.

In the present study, we mitigate these issues by analysing data at a coarser anatomical parcellation of 46 regions, reducing fine-scale spatial spread and limiting leakage between adjacent cortical sources. Source reconstruction and DI estimation were performed separately for each subject and condition using identical pipelines, and statistical contrasts were based on within-subject differences prior to group-level aggregation. Any systematic leakage effects would, therefore, be consistent across states within an individual and are unlikely to account for the differential within-subject patterns observed across anaesthetic conditions.

Importantly, DI does not rely on hyperparameters that weight spatial proximity or amplitude magnitude. Its primary inputs are the estimated VAR coefficients and innovation covariances, derived from regularised least-squares estimation with model order selected by AIC. DI is, therefore, expected to be more sensitive to temporal predictive structure than to spatial amplitude distortions per se.

#### 4.2.2 Sample size, optimisation considerations, and model fitting

The modest sample size warrants replication in larger cohorts. Nonetheless, the cross-condition consistency of the similarity matrices (Figs. 4–7) and the method’s single-subject resolution indicate potential for clinical applications, such as anaesthesia tracking or disorder-of-consciousness assessment, where subject-specific inference is essential.

Although gradient-descent optimisation proved robust, complementary search schemes (e.g., simulated annealing or genetic algorithms) will help clarify whether fragmented emergent landscapes reflect underlying physiology or properties of the optimisation landscape itself.

In the current analysis, we did not explore fitting alternative model classes such as non-linear AR/SS models. However, DI is defined on the state-space representation of the fitted linear multivariate VAR and, therefore, depends on the predictive structure of the estimated model rather than on a specific parametrisation. Future work could compare other model fits which could include those capturing non-linear dynamics and time-varying coefficients in state space models.

#### 4.2.3 Theoretical extensions and future directions

Future work should test whether the macros identified here align with predictions from Integrated Information Theory’s posterior “hot zone” (Albantakis et al., 2023; Boly et al., 2017) or Global Neuronal Workspace Theory’s fronto-parietal broadcast (Dehaene & Changeux, 2011; Dehaene et al., 1998), ideally within adversarial, theory-driven experimental designs (Cogitate Consortium et al., 2023; Melloni et al., 2021).

Augmenting DI with internal diagnostics, including macro self-predictability, model-complexity indices (AIC/BIC), entropy rate, and persistence measures such as spectral radius or autocorrelation decay, will help determine whether candidate *n*-macros are genuinely self-determining rather than only self-containing, or whether they reflect white-noise temporal structure. Such extensions will sharpen the empirical bridge between multiscale emergent structure and conscious experience.

### 4.3 Conclusion

Our study highlights a sweet spot of brain organisation: consciousness is marked by emergent dynamics that are untethered from the lower-level activity yet remain tethered to it across scales. Compared with wake, propofol and xenon push cortical activity towards highly decoupled macrodynamics that also realise a more fragmented emergent dynamical landscape. Whereas wake and ketamine conditions preserve a structured, scale-integrated emergent dynamical landscape despite lower emergence. By extracting these macrodynamics directly from neurophysiological observations, we identify a heterarchical organisation in which scale-integrated emergent structures, rather than fully scale-separable ones, characterise conditions associated with conscious experience. We hope that such results motivate future research to test whether this association causally impacts an individual’s conscious level. The DI framework thus offers a powerful, theory-agnostic lens to map the multiscale dynamical organisation onto cognitive and conscious processing.

## Supplementary Material

Supplementary Material

## Data Availability

The EEG dataset analysed in this study is available in the open-access repository referenced in Sarasso et al. (2015). All relevant scripts, including those for preprocessing, source reconstruction, and dynamical independence analysis, are publicly available via the repositories listed below. Code availability: Custom methods and analysis used in this study are available in the GitHub repository [DI EEG] developed and maintained by B.M.: https://github.com/bmilinkovic/di_eeg. Dynamical independence (DI) analysis was performed using the SSDI toolbox developed by Lionel Barnett: https://github.com/lcbarnett/ssdi. This toolbox depends on the MVGC2 toolbox for Granger causality analysis: https://github.com/lcbarnett/MVGC2.

## Footnotes

1 DI macrovariables may in fact be nested hierarchically (the condition for pure dynamical independence is transitive), but this need not be the case (Barnett & Seth, 2023).

2 The Stiefel manifold $V_{N}\left(n\right)$ is the space of *n*×*N* orthonormal matrices; the closely-related Grassmannian manifold $G_{N}\left(n\right)$ is the space of all *n*-dimensional linear subspaces in an ambient *N*-dimensional Euclidean space. There is a natural (many-to-one) mapping of $V_{N}\left(n\right)$ onto $G_{N}\left(n\right)$, by considering an element of $V_{N}\left(n\right)$ as a (non-unique) orthonormal basis for a subspace in $G_{N}\left(n\right)$.

3 We note that these could be nonlinear, here we restrict ourselves to linear subspaces.
