# Supplementary material for: Emergent multiscale organisation of neural dynamics fragments in anaesthesia
Source: Imaging Neurosci (Camb). 2026 Sep 17;4:IMAG.a.1364. doi: 10.1162/IMAG.a.1364 (PMC13588318; doi:10.1162/IMAG.a.1364)
Supplement: Supplementary file 1 [file IMAG.a.1364_supp.pdf]

Supplementary Materials for: Emergent Multiscale Organisation of Neural Dynamics Fragments in Anaesthesia

Borjan Milinkovic,[1,2,3,4,*] Anil K. Seth,[4,5] Lionel Barnett,[4] Olivia Carter,[1] Thomas Andrillon[2,6]

[1]Melbourne School of Psychological Sciences, University of Melbourne, Melbourne, Australia

[2]INSERM/Institut du Cerveau (ICM), Hôpital de la Pitié-Salpêtrière, Paris, France

[3]Paris-Saclay Institut des Neurosciences, CNRS, Gif-sur-Yvette, Paris, France

[4]Sussex Centre for Consciousness Science, Department of Informatics, University of Sussex, Brighton, UK

[5]Canadian Institute for Advanced Research, Program on Brain, Mind, and Consciousness, Toronto, Canada

[6]Monash Centre for Consciousness & Contemplative Studies, Monash University, Melbourne, Australia

[*]Correspondence: borjan.milinkovic@cnrs.fr

July 1, 2026

1 Supplementary Material

Table 1: Participant-wise Distribution of Epoch Counts across Wakefulness and Anaesthetic Conditions

**Wake**	**Ketamine**	**Propofol**	**Xenon**
155	164	45	169
512	174	506	190
151	150	113	172
184	166	191	152
151	153		150
157			
351			
218			
161			
154			
174			
63			
152			
152			

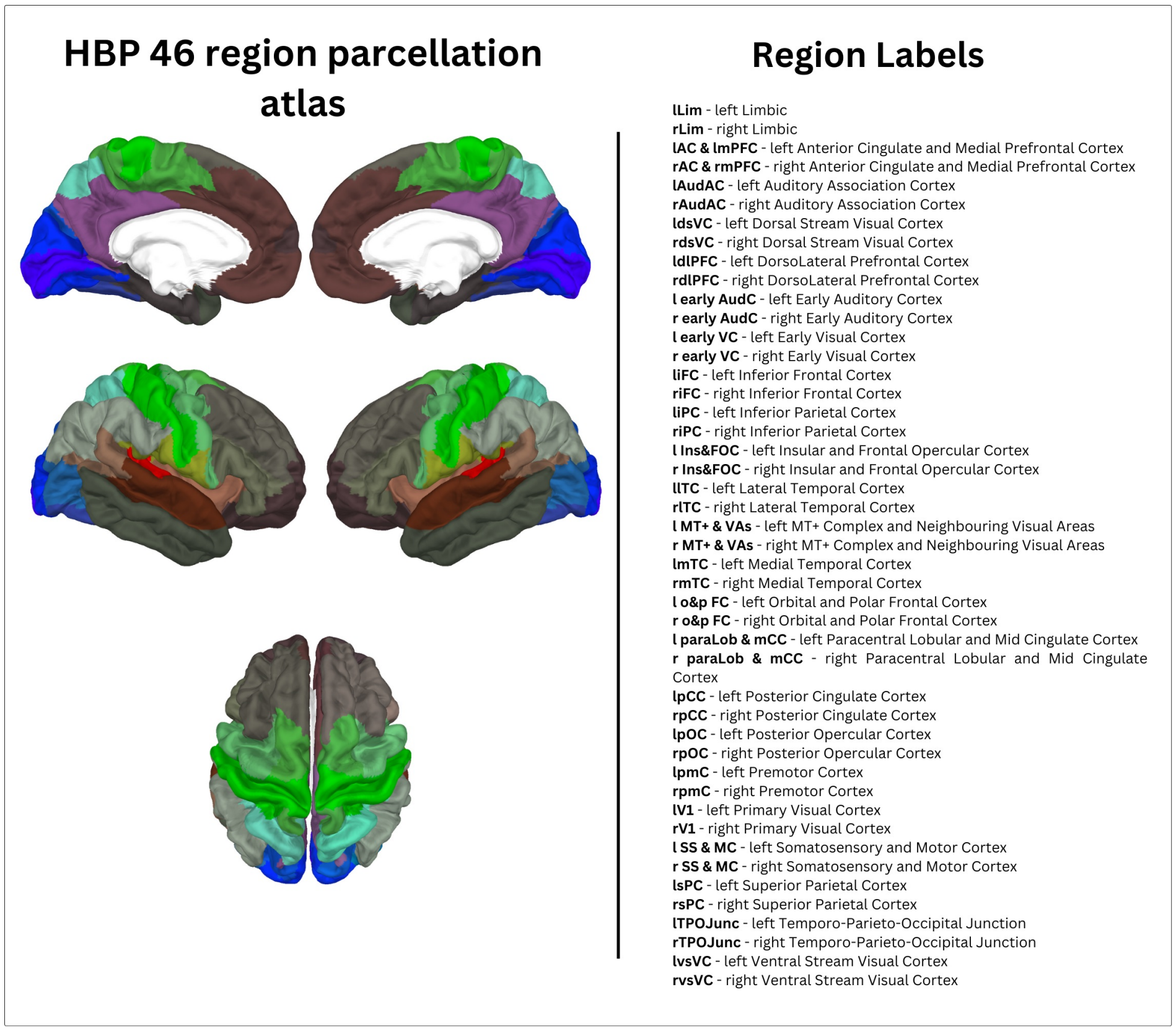

Figure 1: **Reduced HCP Parcellation Atlas.** Used for Source Reconstruction.

Table 2: Conceptual comparison of emergence-related measures.

**Measure**	**What it quantifies**	**What it does not capture**	**Scale-dependent?**	**Identifies macros?**
DI (dynamical closure)	Independence of macroscopic dynamics from their microscopic base (dynamical closure), evaluated across candidate macro dimensionalities	Irreducible joint informational content (synergy) among variables about a target	Yes	Yes
Synergy / PID / O-information	Irreducible joint information among multiple variables about a target beyond individual contributions	Dynamical closure of macroscopic processes across spatial scales	Limited	No
Lempel-Ziv / entropy rate	Signal diversity, unpredictability, or compressibility of time series	Multiscale organisation and predictive independence between macro and micro levels	No	No
Modularity / graph measures	Topological community structure and mesoscale organisation in networks	Dynamical closure (predictive independence) across scales	Implicit only	No

Table 3: Scale-resolved three-agent mixed ANOVA condition $\times$ agent interactions. At each macro scale, condition (Wake vs. Anaesthesia) was treated as a within-subject factor and anaesthetic agent (propofol, xenon, ketamine) as a between-subject factor. The reported term is the condition $\times$ agent interaction. q values are FDR-corrected across the eight macro scales within each metric.

Metric	n-macro	Interaction F	p	q_{FDR}	η_p^2
DD	2	$F(2,11) = 2.72$	.110	.110	.331
DD	3	$F(2,11) = 2.99$	.092	.110	.352
DD	4	$F(2,11) = 2.76$	.107	.110	.334
DD	5	$F(2,11) = 2.75$	.107	.110	.334
DD	6	$F(2,11) = 2.80$	.104	.110	.338
DD	7	$F(2,11) = 2.98$	.092	.110	.352
DD	8	$F(2,11) = 3.09$	.086	.110	.360
DD	9	$F(2,11) = 3.21$	.080	.110	.368
SF	2	$F(2,11) = 1.67$	.232	.412	.233
SF	3	$F(2,11) = 1.54$	.258	.412	.218
SF	4	$F(2,11) = 0.26$	.772	.834	.046
SF	5	$F(2,11) = 0.18$	.834	.834	.032
SF	6	$F(2,11) = 2.22$	.155	.412	.288
SF	7	$F(2,11) = 3.13$	.084	.412	.363
SF	8	$F(2,11) = 2.57$	.121	.412	.319
SF	9	$F(2,11) = 0.81$	.469	.626	.129

Table 4: Summary of scale-resolved pairwise 2×2 mixed ANOVA condition $\times$ agent interactions. q values are FDR-corrected across macro scales within each planned contrast.

Metric	Contrast	p range	q_{FDR} range	Significant n-macros
DD	Propofol vs. Xenon	.893–.997	.997–.997	None
DD	Propofol vs. Ketamine	.120–.154	.154–.154	None
DD	Xenon vs. Ketamine	.018–.028	.028–.028	2, 3, 4, 5, 6, 7, 8, 9
SF	Propofol vs. Xenon	.136–.783	.581–.783	None
SF	Propofol vs. Ketamine	.082–.866	.216–.866	None
SF	Xenon vs. Ketamine	.138–.780	.534–.780	None

Supplementary Text 2: Power Spectral Analysis

We examined the grand-average power spectral density (PSD) across conditions (Fig S2). The results replicate prior findings, including an increase in alpha power (8–13 Hz) under propofol compared to wakefulness (Koskinen et al., 2001; Purdon et al., 2013), a shift toward theta frequencies under ketamine (Purdon et al., 2013), and a flattening of oscillatory peaks in xenon (McGuigan et al., 2021). These spectral characteristics are consistent with previous analyses using the same dataset (Colombo et al., 2019; Sarasso et al., 2015).

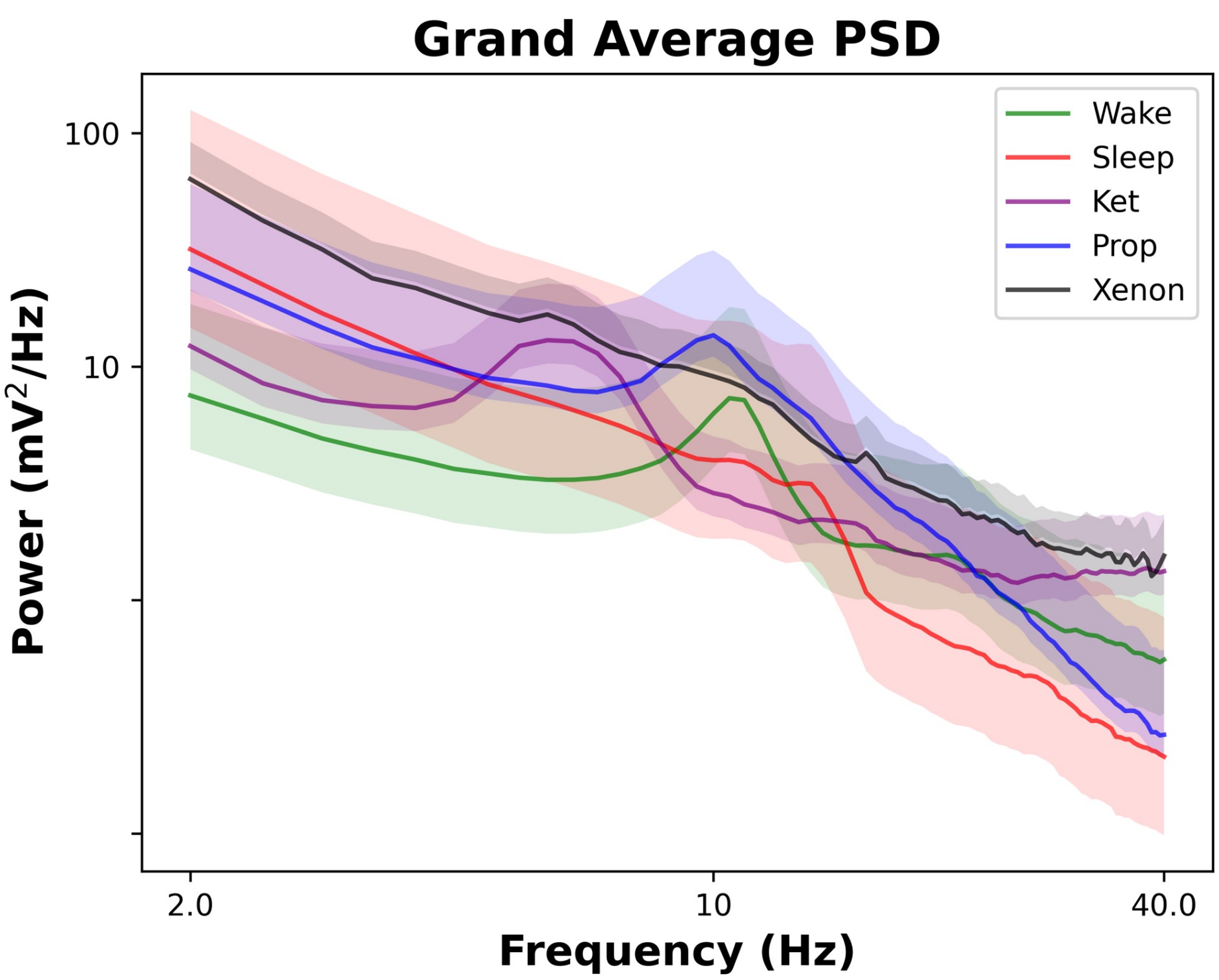

Figure 2: **Grand-average Power Spectral Density across conditions.** PSD estimates with 95% confidence intervals computed from 1,000 bootstrap samples. Ketamine shows a shift of the alpha peak toward lower frequencies, xenon flattens spectral peaks, and propofol increases alpha power.

Supplementary Text 3: Granger Causal Connectivity Matrices

We present grand-average pairwise conditional Granger causality (GC) matrices computed across all source-reconstructed regions for each condition (Fig S3). These matrices capture directed functional connectivity based on pairwise-conditional GC estimates.

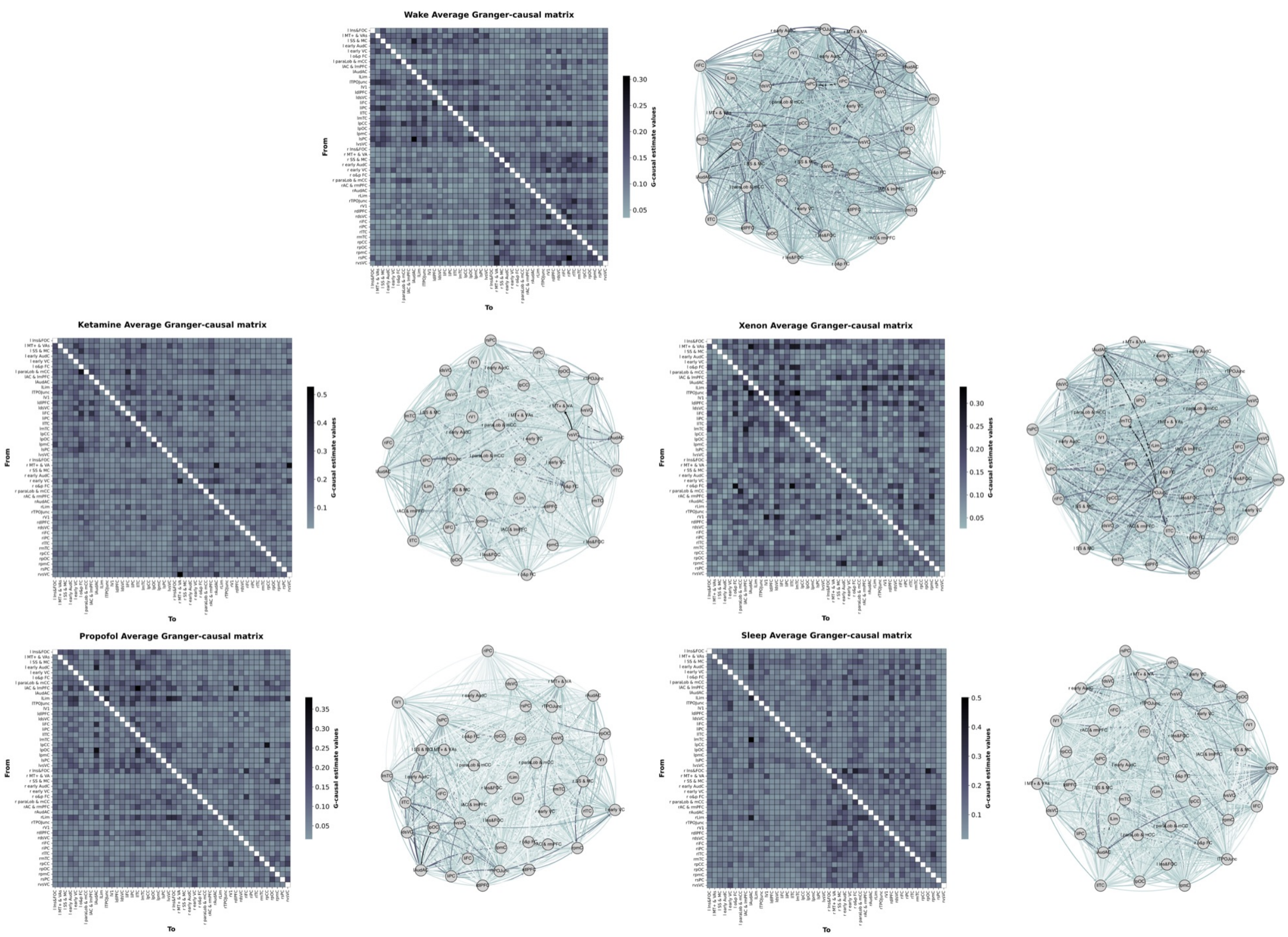

Figure 3: **Granger Causal Connectivity Matrices.** Grand-average pairwise conditional Granger-causality adjacency matrices for all conditions, computed from source-reconstructed EEG using the same regional atlas as shown in the main text.

Supplementary Text 4: Kolmogorov–Smirnov (KS) Tests on n-Macro Similarity Distributions

We assessed the distribution of similarity values between emergent n-macros across optimisation runs and conditions using Kolmogorov–Smirnov (KS) tests (Fig S4). KS statistics compared the empirical cumulative distribution functions (CDFs) of wakefulness against each anaesthetic condition. All conditions yielded significant differences after Bonferroni correction: propofol ($D = 0.39$, $p < 0.0001$), xenon ($D = 0.28$, $p < 0.0001$), and ketamine ($D = 0.11$, $p < 0.001$), indicating meaningful alterations in emergent dynamical structure across states.

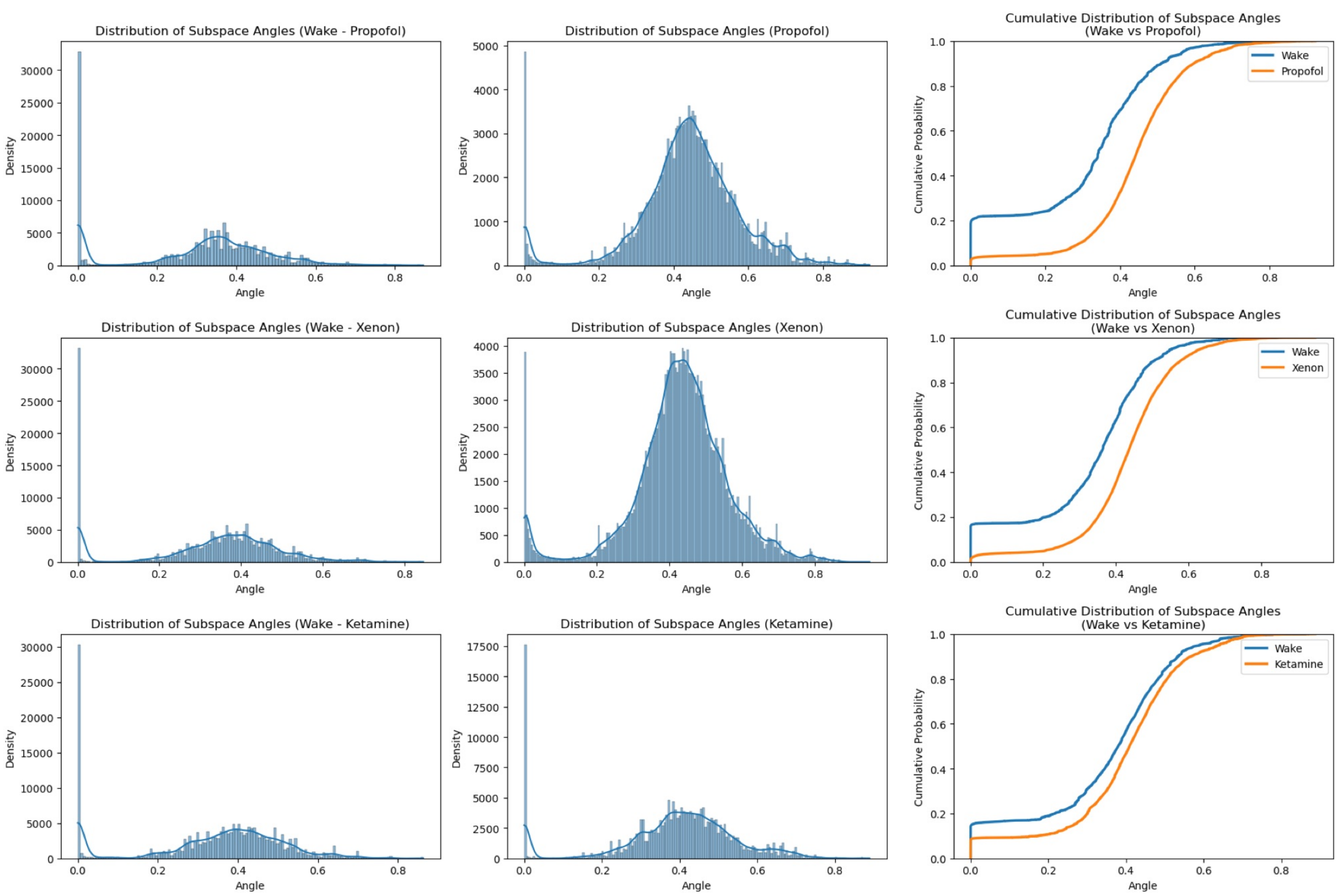

Figure 4: **Distributions of n-Macro Similarity and KS Test Results.** Empirical CDFs of n-macro similarity scores for wakefulness and each anaesthetic condition. KS tests reveal significantly different distributions between wakefulness and all anaesthetic conditions, indicating disrupted or fragmented macroscopic structure.

Supplementary Text 5: Explanation of Subject-Level Regional Contribution Maps of Significant Regional Contributions

To complement the group-level statistical analyses presented in the main text, we provide here individual participant-level maps of significant regional contributions to the optimised macroscale variables (n-macros) across Wake and each anaesthetic condition (Propofol, Ketamine, Xenon). These maps are intended to give a more detailed spatial perspective on how emergent macroscale structure reorganises under each anaesthetic, and to illustrate the degree of within-subject and between-subject variability across macroscales.

For each subject, we show results across macroscales 2–9, as well as an aggregate union map summarising regions that were significant at any scale. Regions are colour-coded to indicate whether they show significant participation in Wake, in the anaesthetic condition, or in both. These complementary visualisations provide an intuitive view of the spatial patterns underlying the quantitative measures reported in the main figures.

Supplementary Text 6: Wake vs Propofol — Significant Regional Contributions

In this section we provide individual participant-level maps showing the significant cortical regions contributing to the optimised macroscale variables (n-macros) for Wake vs Propofol. For each participant and each macroscale (2–9), regions are colour-coded according to whether they show significant participation in Wake only, Propofol only, or both. An aggregate union map ("Any Macroscale") shows regions that were significant at any tested scale.

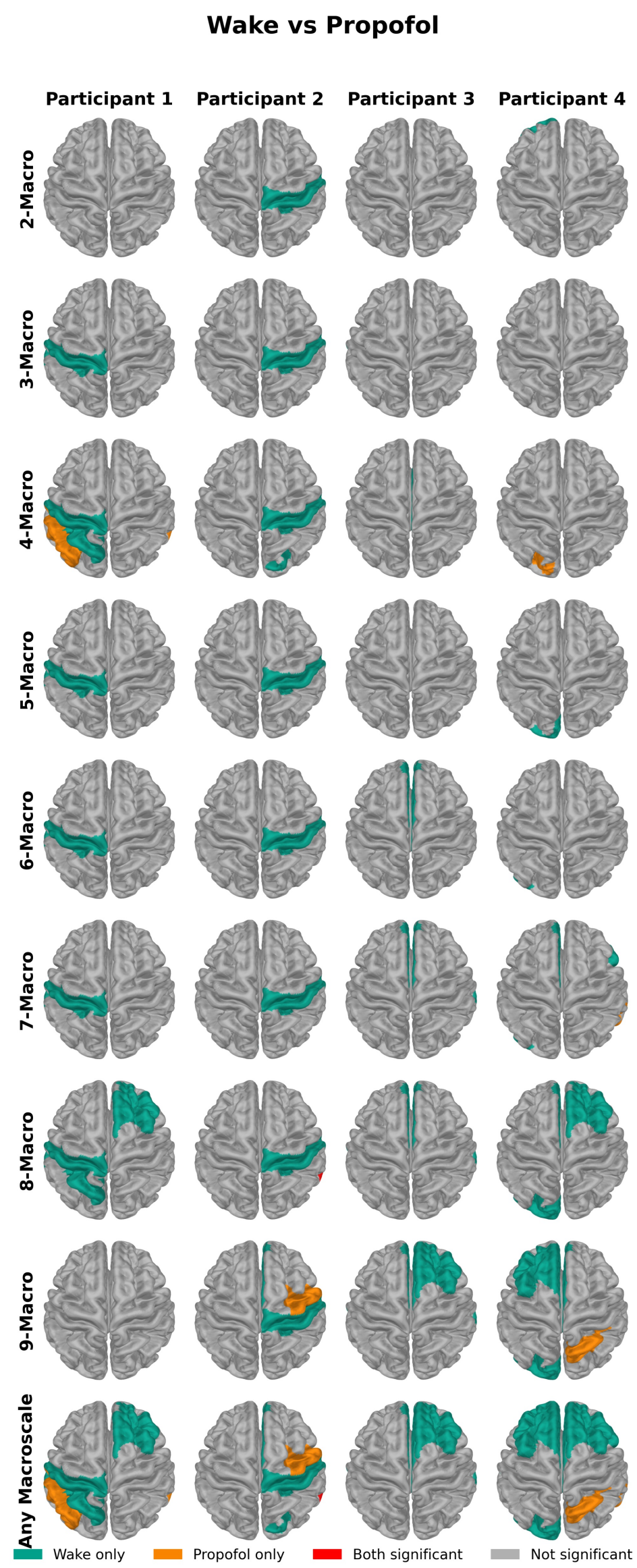

Figure 5: **Wake vs Propofol:** significant regional contributions to optimised macroscales (2–9) and union map ("Any Macroscale") for each participant (N=4). Green = Wake only; Orange = Propofol only; Red = both; Grey = non-significant.

Supplementary Text 7: Wake vs Xenon — Significant Regional Contributions

Here we display participant-level maps for Wake vs Xenon, again showing significant regional participation in the optimised macroscales across individual subjects. The union map highlights regions consistently participating at any scale. These results reveal widespread and often bilateral changes in macroscale structure under xenon anaesthesia.

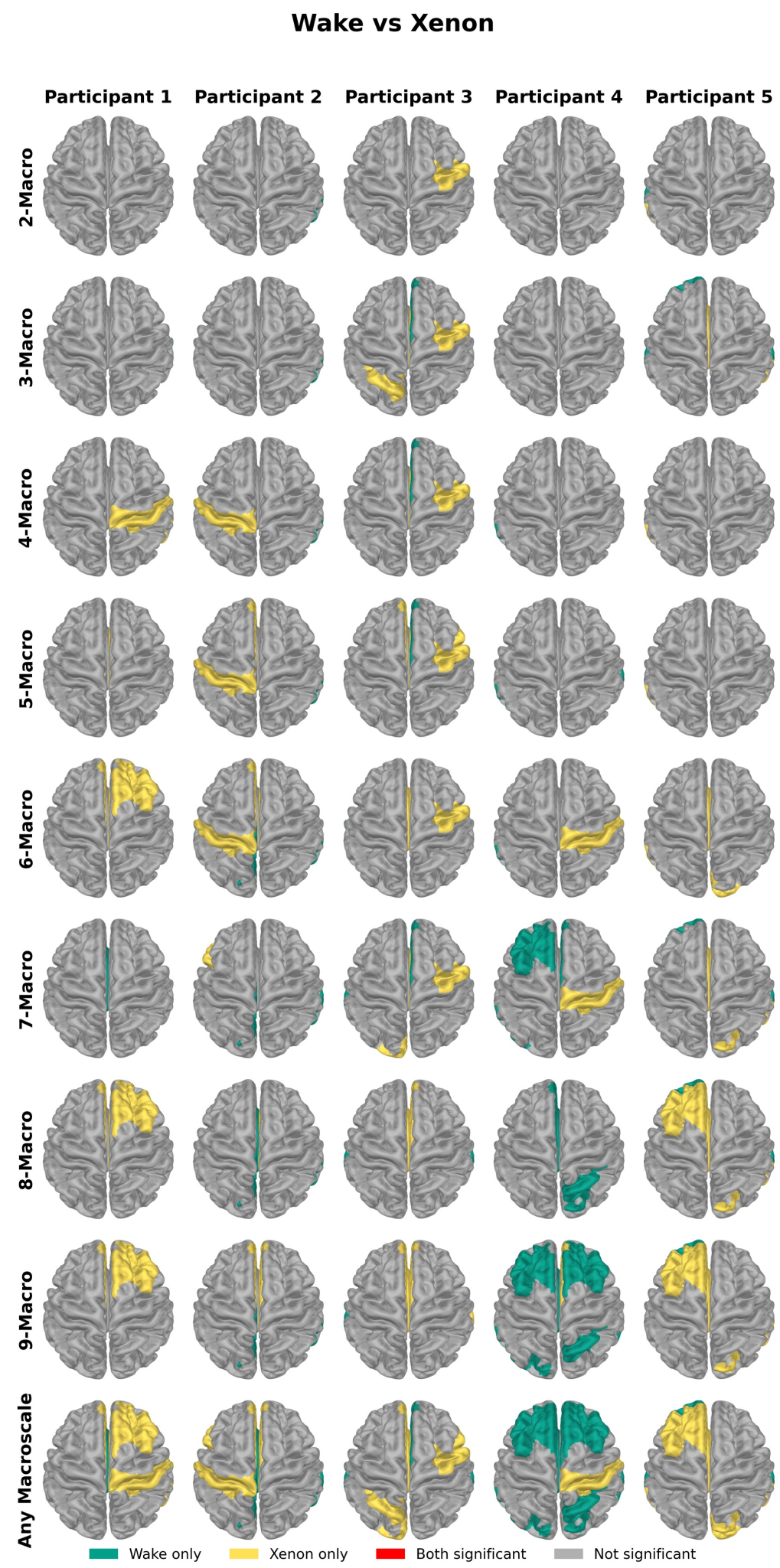

Figure 6: **Wake vs Xenon:** significant regional contributions to optimised macroscales (2–9) and union map ("Any Macroscale") for each participant (N=5). Green = Wake only; Yellow = Xenon only; Red = both; Grey = non-significant.

Supplementary Text 8: Wake vs Ketamine — Significant Regional Contributions

This section presents individual participant-level maps for Wake vs Ketamine. The same conventions are used as for the previous figure: regions significantly contributing to the optimised macroscales are displayed for each participant and each scale, with a union map summarising robust contributions across scales. These maps illustrate the heterogeneous and spatially distributed changes in macroscale participation patterns under ketamine.

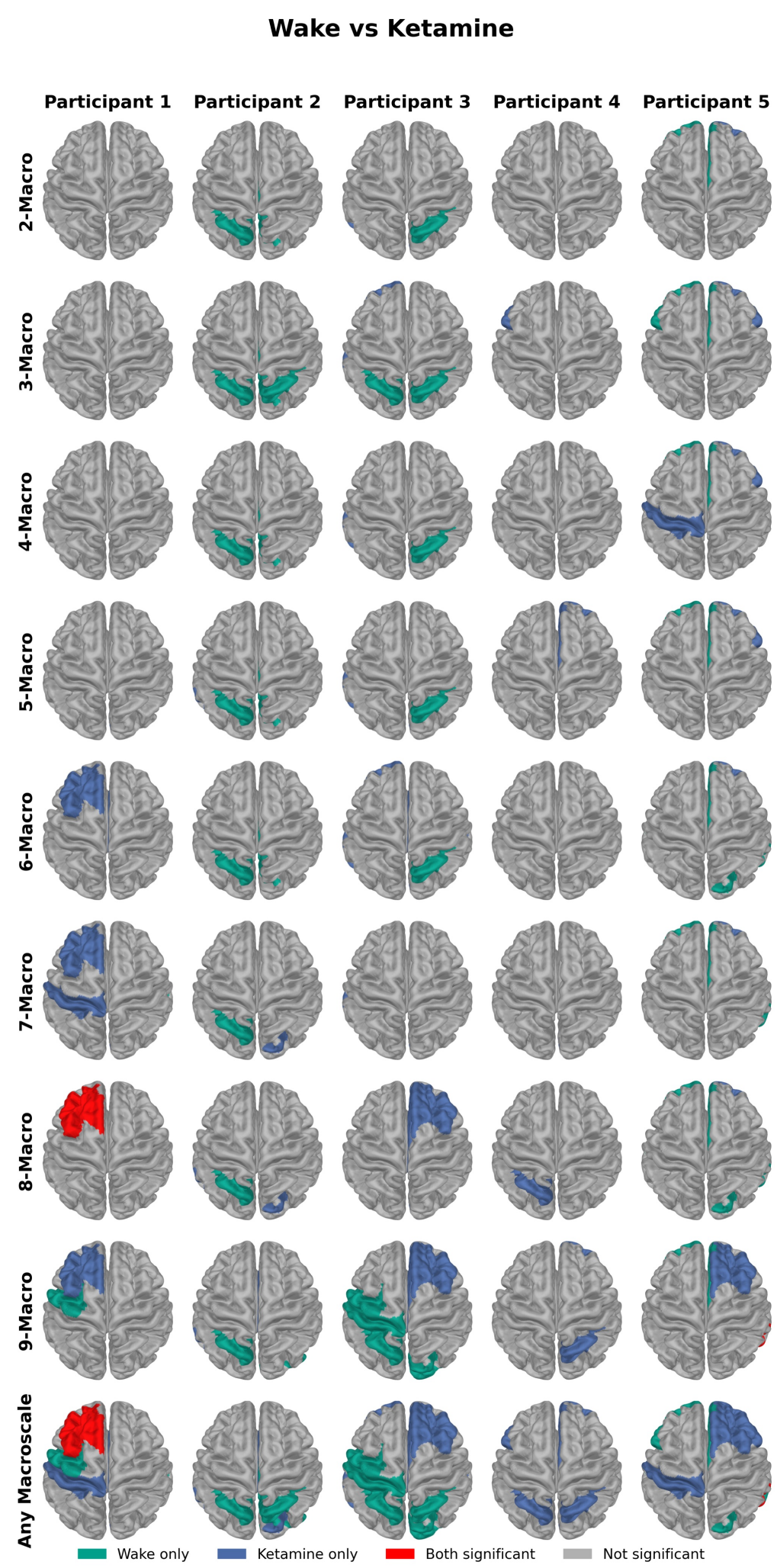

Figure 7: **Wake vs Ketamine:** significant regional contributions to optimised macroscales (2–9) and union map ("Any Macroscale") for each participant (N=5). Green = Wake only; Blue = Ketamine only; Red = both; Grey = non-significant.

References

Colombo, M. A., Napolitani, M., Boly, M., Gosseries, O., Casarotto, S., Rosanova, M., Brichant, J.-F., Boveroux, P., Rex, S., & Laureys, S. (2019). The spectral exponent of the resting eeg indexes the presence of consciousness during unresponsiveness induced by propofol, xenon, and ketamine. *NeuroImage*, *189*, 631–644.

Koskinen, M., Sepṕanen, T., Tuukkanen, J., Yli-Hankala, A., & J́antti, V. (2001). Propofol anesthesia induces phase synchronization changes in eeg. *Clinical neurophysiology*, *112*(2), 386–392.

McGuigan, S., Evered, L., Silbert, B., Scott, D. A., Cormack, J. R., Devapalasundaram, A., & Liley, D. T. (2021). Comparison of the spectral features of the frontal electroencephalogram in patients receiving xenon and sevoflurane general anesthesia. *Anesthesia & Analgesia*, *133*(5), 1269–1279.

Purdon, P. L., Pierce, E. T., Mukamel, E. A., Prerau, M. J., Walsh, J. L., Wong, K. F. K., Salazar-Gomez, A. F., Harrell, P. G., Sampson, A. L., Cimenser, A., et al. (2013). Electroencephalogram signatures of loss and recovery of consciousness from propofol. *Proceedings of the National Academy of Sciences*, *110*(12), E1142–E1151.

Sarasso, S., Boly, M., Napolitani, M., Gosseries, O., Charland-Verville, V., Casarotto, S., Rosanova, M., Casali, A. G., Brichant, J. F., Boveroux, P., Rex, S., Tononi, G., Laureys, S., & Massimini, M. (2015). Consciousness and complexity during unresponsiveness induced by propofol, xenon, and ketamine. *Current Biology*, *25*(23), 3099–3105. https://doi.org/10.1016/J.CUB.2015.10.014/ATTACHMENT/0C5300ED-17C7-4442-862A-D16AA3D56E32/MMC1.PDF
